# Expression Analysis and Functional Characterization of *CER1* Family Genes Involved in Very-Long-Chain Alkanes Biosynthesis in *Brachypodium distachyon*

**DOI:** 10.3389/fpls.2019.01389

**Published:** 2019-11-01

**Authors:** Hongqi Wu, Shandang Shi, Xiaoliang Lu, Tingting Li, Jiahuan Wang, Tianxiang Liu, Qiang Zhang, Wei Sun, Chunlian Li, Zhonghua Wang, Yaofeng Chen, Li Quan

**Affiliations:** ^1^State Key Laboratory of Crop Stress Biology for Arid Areas, College of Agronomy, Northwest A&F University, Yangling, China; ^2^Shaanxi Province Tobacco Company of China National Tobacco Company, Xi’an, China

**Keywords:** wax, alkanes, *CER1*, expression, drought stress, *Brachypodium distachyon*

## Abstract

Cuticular wax accumulation and composition affects drought resistance in plants. *Brachypodium distachyon* plants subjected to water deficit and polyethylene glycol treatments resulted in a significant increase in total wax load, in which very-long-chain (VLC) alkanes were more sensitive to these treatments than other wax compounds, implying that VLC alkanes biosynthesis plays a more important role in drought resistance in *B. distachyon*. *ECERIFERUM1* (*CER1*) has been reported to encode a core enzyme involved in VLC alkanes biosynthesis in Arabidopsis (*Arabidopsis thaliana*), but few corresponding genes are investigated in *B. distachyon*. Here, we identified eight *CER1* homologous genes in *B. distachyon*, namely *BdCER1-1* to *BdCER1-8*, and then analyzed their sequences feature, expression patterns, stress induction, and biochemical activities. These genes had similar protein structure to other reported *CER1* and *CER1*-like genes, but displayed closer phylogenetic relationship to the rice *OsGL1* genes. They were further found to exhibit various tissue expression patterns after being induced by abiotic stresses. Among them, *BdCER1-8* gene showed extremely high expression in leaves. Heterologous introduction of *BdCER1-8* into the Arabidopsis *cer1* mutant rescued VLC alkanes biosynthesis. These results indicate that *BdCER1* genes are likely to be involved in VLC alkanes biosynthesis of *B. distachyon*. Taken together, *BdCER1-8* seems to play an explicit and predominant role in VLC alkanes biosynthesis in leaf. Our work provides important clues for further characterizing function of *CER1* homologous genes in *B. distachyon* and also an option to improve drought resistance of cereal crops.

## Introduction

The tissue surfaces of higher plants are covered by cuticle consisting of cutin and cuticular wax (Kerstiens, 2006; Pollard et al., 2008; Li and Beisson, 2009). As the major structural constituent in the cuticle, cutin is covered or embedded by cuticular wax (Pollard et al., 2008; Li and Beisson, 2009). Cuticular wax is a complex mixture of the very-long-chain fatty acids (VLCFAs, ≥C20) and their derivatives, such as primary alcohols, aldehydes, alkanes, alkenes, branched alkanes, secondary alcohols, unsaturated fatty alcohols, ketones, wax esters, and cyclic compounds (Samuels et al., 2008; Lee and Suh, 2013). In addition to its primary function in restraining uncontrolled non-stomatal water loss in plants, the cuticle also plays roles in helping plants against excess UV radiation, insects, and bacterial and fungal pathogens (Eigenbrode and Espelie, 1995; Solovchenko and Merzlyak, 2003; Shepherd and Grifﬁths, 2006). Surprisingly, an unexpected function of wax was found to participate in proper pollen–stigma interaction (Preuss et al., 1993).

Cuticular wax biosynthesis occurs in epidermal cells (Kunst and Samuels, 2009). First, the *de novo* synthesis of C16 or C18 fatty acid is processed in the plastid of epidermal cells, which are catalyzed into C16 or C18 fatty acyl-CoAs by the fatty acid synthase complex. Then these products are elongated to VLCFAs mainly with 26 to 32 carbons by fatty acid elongase complex in the endoplasmic reticulum (Samuels et al., 2008; Lee and Suh, 2013; Kunst and Samuels, 2009). After elongation, VLCFAs are converted into primary alcohols and wax esters by the alcohol-forming pathway or alkanes and their derivatives by the alkane-forming pathway, respectively (Samuels et al., 2008; Kunst and Samuels, 2009). Among these wax components, VLC alkanes are predominant in Arabidopsis, accounting for 50–80% of the total wax contents (Bourdenx et al., 2011). They are increased by 93% under water deficit, suggesting that VLC alkanes biosynthesis plays an important role in drought resistance (Kosma et al., 2009).

Several genes involved in VLC alkanes biosynthesis have been identified through the phenotypic characterizations of the Arabidopsis *cer1* and *cer3* mutants. Compared with the wild-type plants, the *cer1* mutant displays glossy green stems, significantly decreased contents of alkanes and their derivatives, and slightly increased contents of aldehydes, indicating that *CER1* encodes an aldehyde decarboxylase responsible for the conversion of aldehydes to alkanes (Aarts et al., 1995; Bourdenx et al., 2011). In the *cer3* mutant, the stems also exhibit glossy green, but the contents of alkanes, secondary alcohols, ketones, and aldehydes go decreased dramatically, suggesting that *CER3* encodes an enzyme critical for production of aldehydes using VLC acyl-CoAs as substrates (Chen et al., 2003; Kurata et al., 2003; Rowland et al., 2007). Further, Bernard et al. (2012) used yeast as a heterologous expression system to build a new pathway of VLC alkanes biosynthesis: CER1 physically interacts with CER3 and CYTB5s to convert VLC acyl-CoAs to VLC alkanes, during which CER1/CER3 complex is the core component of this process, while CYTB5s function to improve CER1 efficiency as CER1-specific cofactors (Bernard et al., 2012). As another member of the *CER1* family in Arabidopsis, *CER1-like1* also plays a vital role in VLC alkanes biosynthesis by using the same mechanism as that of *CER1*, but the CER1-like1/CER3 complex prefers to produce shorter chain alkanes than the CER1/CER3 complex, suggesting that alkane-forming complexes have distinct chain-length specificities and are coexisted in plants (Pascal et al., 2019).

The biological functions of *CER1* and *CER3* have been well characterized in Arabidopsis. The *CER1* gene was found to be mainly expressed in the epidermis of aerial organs and could also be induced by abiotic stresses (Bourdenx et al., 2011). T-DNA insertional mutants of *CER1* are devoid of VLC alkanes and their derivatives, whereas the contents of odd carbon-numbered alkanes and iso-branched alkanes are significantly increased in the *CER1*-overexpressing plants (Bourdenx et al., 2011). The *cer1* mutant exhibits male sterility under low humidity, whereas *CER1*-overexpressing plants show reduced cuticle permeability, slow response to soil water deficit, and sensitive response to bacterial and fungal pathogens (Bourdenx et al., 2011). On the other hand, *CER3* transcript is detected in silique, leaf, stem, and flower, but is absent in root. Loss-of-function mutation in *CER3* alters cuticle membrane and wax synthesis. Moreover, the *cer3* mutant shows post-genital organ fusions, decreased fertility under low humidity, and reduced stomatal index (Chen et al., 2003; Rowland et al., 2007). A recent study shows that mutation of *CER1* or *CER3* can also reduce the amounts of branched alkanes in leaf and flower, implying that they may partially be involved in branched chain wax component synthesis as well (Busta and Jetter, 2017).

Several orthologs of *CER1* and *CER3* genes have also been identified in other plants. As a homologous gene of *CER1*, *BnCER1* transcript is most severely suppressed among wax biosynthetic genes in a glossy mutant (*BnaA.GL*) of *Brassica napus*, suggesting that *BnCER1* may be a candidate gene involved in alkane-forming pathway (Pu et al., 2013). *CsCER1* and *CsWAX2* in Cucumber (*Cucumis sativus*) are respective homologs of *CER1* and *CER3*, both of which are involved in VLC alkanes biosynthesis and affect pollen fertility (Wang et al., 2015a, Wang et al., 2015b). The maize (*Zea mays*) *CER3* homologous gene *GLOSSY1* (*GL1*) is involved in cuticular wax accumulation in seedling leaves (Sturaro et al., 2005). A BLAST search in rice database using GL1 protein further retrieved 11 putative *GL1* paralogous genes, named *OsGL1-1* to *OsGL1-11* (Islam et al., 2009). Among them, *OsGL1-1*, *OsGL1-2*, and *OsGL1-3* are more closely related to *CER3* and *GL1*, while *OsGL1-4*, *OsGL1-5*, *OsGL1-6*, and *OsGL1-7* are more closely related to *CER1* (Islam et al., 2009). Despite of their large family members, these *CER1* and *CER3* homologs in rice exhibit partially divergent functions during plant growth, pollen development, and cuticular wax biosynthesis. *OsGL1-1* was found to be expressed ubiquitously, involved in leaf cuticular wax and cuticle membrane formations in rice (Qin et al., 2011). *OsGL1-2*, *OsGL1-3*, and *OsGL1-6* show varying expression levels in various organs and the disruption of their functions result in different wax productions and drought resistance levels (Islam et al., 2009; Qin et al., 2011; Zhou et al., 2013; Zhou et al., 2015). As homologs of *CER1, Wax-deficient Anther 1* (*WDA1*) and *OsCER1* affect wax production and pollen development in rice (Jung et al., 2006; Ni et al., 2018). However, as an outstanding referencing model plant for studying functional genomics of grasses and cereals such as wheat (*Triticum aestivum* L.) and barley (*Hordeum vulgare* L.) (Draper et al., 2001; Vogel et al., 2010), *Brachypodium distachyon* is still a bit far from being well investigated on VLC alkanes biosynthesis. Only few studies have been carried out including one recent report showing that *BdWAX2* is required for cuticular wax biosynthesis (Hsia et al., 2017). But the alkane-forming pathway in *B. distachyon* remains unknown. To address this concern, a full picture of the *CER1* homologs involved in VLC alkanes biosynthesis in *B. distachyon* need to be investigated.

In this article, we systematically examined the influence of drought stress on leaf cuticular wax of *B. distachyon*, and found that VLC alkanes may play a critical role against drought stress. Then, sequence features and expression profiles of *CER1* homologous genes that are possibly involved in VLC alkanes biosynthesis in *B. distachyon* were analyzed in details. Further, we generated *BdCER1-8* heterologous expression in the Arabidopsis *cer1* mutant to unveil its role in wax biosynthesis. Our results show that VLC alkanes are more sensitive to drought stress than other wax compounds. It was found that there were eight *CER1* homologous genes identified in *B. distachyon* that were likely involved in VLC alkanes biosynthesis. Among them, *BdCER1-8* rescued wax production defect in the *cer1* mutant by participating in the VLC alkanes biosynthesis.

## Materials and Methods

### Plant Growth and Treatments

*B. distachyon* ‘Bd21’ seeds were germinated and grown in the greenhouse with a 14-h light/10-h dark cycle. Two-month-old seedlings were used for all stress treatments. For water deficit treatment, plants were stopped watering until lower leaves were wilted, which needed 6 days. For polyethylene glycol (PEG) treatment, plants were watered with 20% (w/v) PEG6000 three times over a 6-day period. Plants watered as needed with tap water were used as control plants. All leaves were harvested at seventh day after treatments for extracting cuticular wax. To investigate the effects of stresses on the expressions of *BdCER1* genes, 2-month-old seedlings were incubated in water for 2 days and then transferred to water containing 200 mM NaCl, 20% (w/v) PEG6000, or 100 µM ABA. For drought stress evaluation, surface water of roots was dried gently by napkins, and then seedlings were transferred to filter papers without water supply. Plant leaves under all treatments were sampled at 0, 2, 4, 6, 8, 12, and 24 h. To examine tissue-specific expression profiles of the *BdCER1* genes, four tissues (leaves, stems, nodes, and roots) were collected.

Seeds of T3 homozygous *BdCER1-8* transgenic lines, the *cer1* mutant (SALK_008544) and wild-type Arabidopsis Col-0 (WT) plants were germinated on MS medium. After 2 weeks, seedlings were transferred into Arabidopsis seedling substrate with moderate vermiculites and grown in the illumination incubator at 23°C, 14-h-light/10-h-dark photoperiod. Two-month-old inflorescence stems and 4-week-old rosette leaves were collected to analyze the expression level of *BdCER1-8*, wax crystal, and cuticular wax, respectively. Four-week-old rosette leaves were further used to check chlorophyll efflux and water loss rates. For soil water deficit experiment, 4-week-old different lines were deprived of water until the *cer1* mutant wilted. All materials for expression analysis were immediately frozen in liquid nitrogen and stored at −80°C for further analysis.

### Identification and Sequence Analyses of *CER1* Homologous Genes in *B. distachyon*

To identify putative *CER1* homologous genes in *B. distachyon*, the CER1 protein sequence (GenBank accession: NP_001184890) was subjected to do BLASTP search against *B. distachyon* genome by using the Plant Genomics Resource database (https://phytozome.jgi.doe.gov/). All putative non-redundant protein sequences were manually examined with Pfam program (http://pfam.xfam.org/search) to confirm the presence of the fatty acid hydroxylase (FAH) superfamily (accession no. PF04116). Finally, these potential protein sequences that harbor the FAH superfamily were used as candidate members of CER1 homologous proteins and were further analyzed. The reported *CER1* homologous genes in Arabidopsis, cucumber, rape, rice, and maize were obtained from the NCBI database. All sequences of CER1-homologous proteins were listed in **Supplementary Data Set 1**.

The amino acid sequences of CER1 homologous proteins were aligned by ClustalW and then a phylogenetic tree was constructed using MEGA5 software based on the neighbor-joining method (Saitou and Nei, 1987; Tamura et al., 2011). The molecular weight (MW) and theoretical isoelectric point (pI) of BdCER1s were obtained using the ProtParam tool (http://web.expasy.org/protparam/) (Bjellqvist et al., 1994). The potential motifs in protein sequences were analyzed using the MEME program (http://meme-suite.org/tools/meme) (Bailey et al., 2009). To confirm whether the FAH superfamily of BdCER1 proteins has three His-rich motifs or not, the amino acid sequences of FAH superfamily from different plants were aligned by using ClustalX program with default parameters (Thompson et al., 1997).

### Cuticular Wax Extraction and Chemical Characterization

Bd21 leaves and Arabidopsis leaves or stems were immersed in chloroform for 60 s to extract cuticular wax, respectively. N-Tetracosane (C24) was used as an internal standard in each sample. Each sample was transferred to GC vials and dried under nitrogen gas, and then they were derivatized with 30 µl pyridine (Alfa Aesar) and 30 µl BSTFA [bis-N,O-(trimethylsilyl) trifluoroacetamide] (Fluka) at 70°C for 60 min. After derivatization, all samples were dried again under nitrogen gas, and re-dissolved in 500 µl chloroform. Gas chromatography–mass spectrometry (GC–MS) (GCMS-QP2010, SHIMADZU, Japan) and GC with flame ionization detector (GC-FID) (GC-2010Plus, SHIMADZU, Japan) were used to do qualification and quantification analysis, respectively. The surface areas were calculated using digital images and ImageJ software. The wax contents were expressed per unit of surface area.

### RNA Extraction and Transcript Level Analysis

The total RNA was extracted using the HiPure Plant RNA Mini Kit (Magen, Guangzhou, China) following standard protocol. Before reverse transcription, about 1–2 µg total RNA was treated with gDNA Eraser to remove genomic DNA, and then the first-strand cDNA was synthesized by PrimeScript RT Enzyme Mix I (Takara, Dalian, China) according to the manufacturer’s instruction. Finally, the products were diluted 10-fold with sterile distilled water.

Quantitative real-time PCR (qRT-PCR) was carried out by using an ABI StepOnePlus instrument (Applied Biosystems). Each reaction volume of 25 µl contained 12.5 µl SYBR^®^ Green I Mix (ToYoBo, Osaka, Japan), 2.5 µl cDNA, 1 µl 10 µM forward primer, 1 µl 10 µM reverse primer, and 8 µl ddH_2_O. The PCR cycle used was as follows: 95°C for 1 min, 40 cycles of 95°C for 15 s, 58°C for 30 s, and 72°C for 45 s. Data were collected during the extension step. Dissociation curve analysis was performed as follows: 95°C for 15 s, 60°C for 1 min, and 95°C for 15 s. Specific primers of *BdCER1* genes were listed in **Supplementary Table 7**. *BdACTIN* gene (Bradi_1g10630) was selected as an endogenous control. To check expression levels of *BdCER1-8* in leaves and stems of different transgenic Arabidopsis lines, both semi-quantitative RT-PCR and qRT-PCR assays were performed using *AtACT8* gene (At1g49240) as an endogenous control (corresponding primers also listed in **Supplementary Table 7**). The relative expression levels were analyzed using the 2^−ΔΔCT^ method (Livak and Schmittgen, 2001). Expression levels of the *BdCER1* homologous genes under abiotic stresses were analyzed using the software package of Heatmap on the BMKCloud platform (www.biocloud.net).

### Arabidopsis Transformation

The full-length CDS of *BdCER1-8* was isolated from leaves of 2-month-old plants using gene-specific primers (**Supplementary Table 7**) and cloned into pCXSN vector under the control of 35S promoter. The construct was introduced into *Agrobacterium tumefaciens* strain GV3101 by freeze–thaw method. Two *cer1* mutant lines: *cer1-1* (SALK_008544) and *cer1-2* (SALK_014839) have been previously characterized in Bourdenx et al. (2011). The full-length transcript of *CER1* is not detected in the *cer1-1* allele, but a weak *CER1* band can still be detected in the *cer1-2* allele by RT-PCR (Bourdenx et al., 2011), which may interfere the transgene effect. Thus, as a null-allele and due to its severest phenotypes among all alleles, we picked *cer1-1* (SALK_008544) as the background material for examining the transgene effect of *BdCER1-8* following the Arabidopsis transformation protocol. The *cer1-1* (SALK_008544) seeds were obtained from the Arabidopsis Biological Resource Centre (ABRC). As a result after transformation, we obtained over 30 T1 independent transgenic lines that exhibited various levels of transgene expression and morphological phenotypes. Genomic DNAs were extracted from the rosette leaves of transgenic plants, and PCR was performed to confirm positive transformants by standard PCR protocol using specific primers (**Supplementary Table 7**). qPCR was further carried out to examine the relative *BdCER1-8* expression among all individual positive transformants. As a result, 11 ones with the highest expressions were chosen for further selection of *BdCER1-8* transgene homozygotes on MS medium containing 50 mg/L hygromycin. The resulting homozygotes in T3 generation coming from independent T1 lines were retained for subsequent analyses.

### Phenotyping and Measurements of Transgenic Plants

Chlorophyll efflux, water loss, and soil water deprivation experiments were performed to measure the influence of *BdCER1-8* overexpression on cuticle permeability in the *cer1* mutant. These three assays were conducted as described previously (Kosma et al., 2009). To investigate the influence of *BdCER1-8* overexpression on wax crystal formation, scanning electron microscopy (SEM) was conducted as described previously (Wang et al., 2017).

## Results

### Changes in Cuticular Wax Accumulation of *B. distachyon* Leaves Under Drought Stress

**Figure 1:**
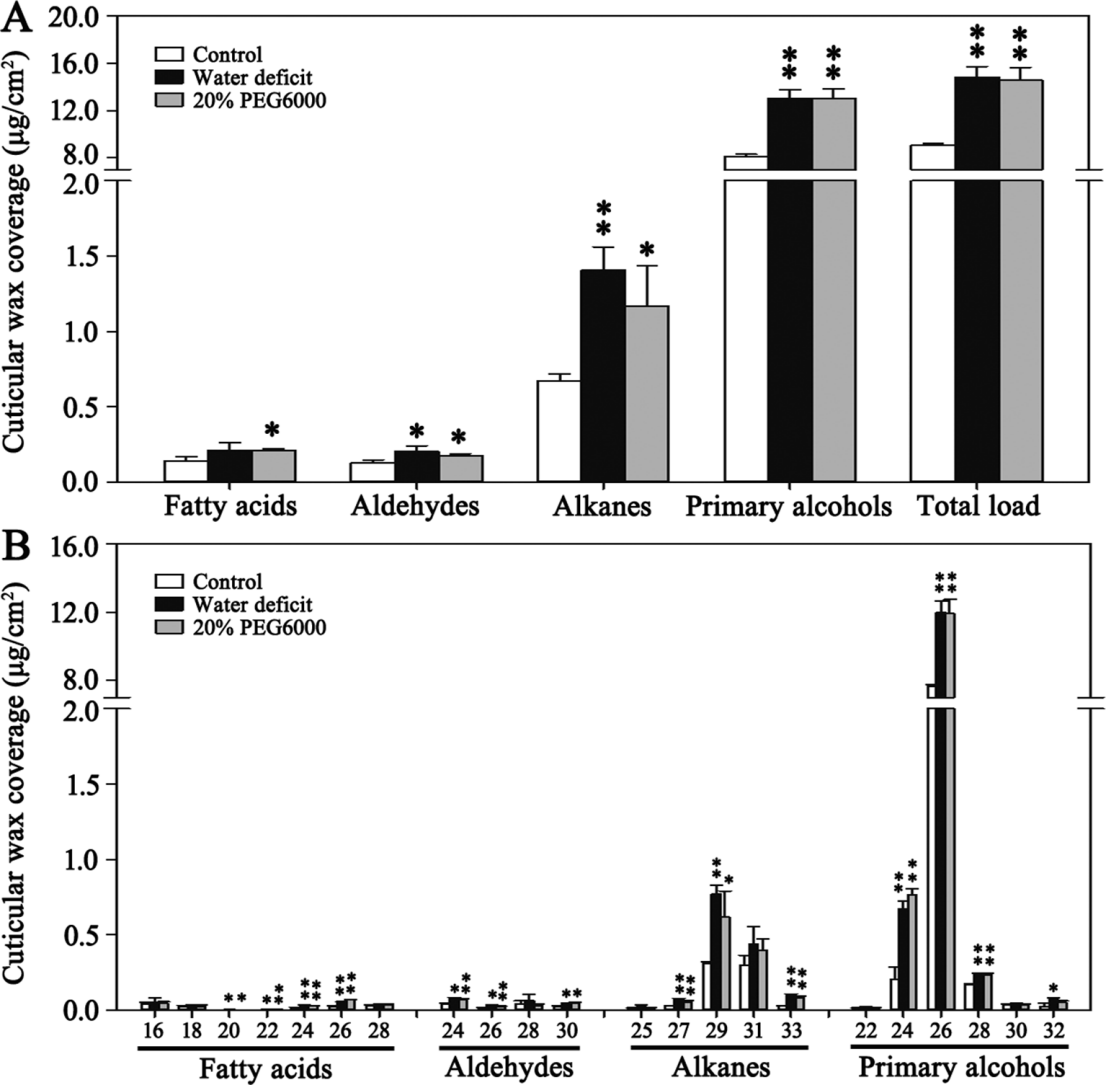
Cuticular wax accumulation and chain length distribution of the individual wax constituents on Bd21 leaves under various treatments. **(A)** Changes in wax components and total load under various treatments. **(B)** Changes in chain length of the individual wax constituents under various treatments. The results show averages of three biological replicates, and error bars indicate ± SD. Asterisks indicate significant differences from control (t-test: * for p < 0.05; ** for p < 0.01). Numbers on the x-axis of **(B)** indicate carbon numbers of the compounds.

Cuticular wax of *B. distachyon* ‘Bd21’ leaves under water deficit and 20% PEG6000 treatments were measured to assess the effect of drought stress on wax composition. In the control leaves, total wax content was 9.04 µg/cm^2^, which was increased by 64.0% to 14.83 µg/cm^2^ under water deficit treatment and by 61.1% to 14.57 µg/cm^2^ with 20% PEG6000 treatment, respectively (**Figure 1A**, **Supplementary Table 1**). Compared to the control leaves, the primary alcohols content under the water deficit and 20% PEG6000 treatments was increased by 60.5% and 60.6%, respectively (**Figure 1A**, **Supplementary Table 1**). The alkanes content was increased by 108.9% and 73.6%, respectively (**Figure 1A**, **Supplementary Table 1**). The aldehydes content was increased by 61.7% and 39.7%, respectively (**Figure 1A**, **Supplementary Table 1**). The fatty acids content was increased by 50.4% and 50.0%, respectively (**Figure 1A**, **Supplementary Table 1**). However, wax composition was unchanged by all treatments, but the contents of different chain length wax components exhibited different elevation levels (**Figure 1B**). Significant increases in primary alcohols C24 (223.8% and 267.5%), C26 (56.4% and 55.7%), C28 (39.8% and 39.3%), and C32 (172.3% and 102.2%); alkanes C27 (164.4% and 107.3%), C29 (150.9% and 101.0%), and C33 (271.8% and 196.3%); aldehydes C24 (69.0% and 66.8%), C26 (84.2% and 62.6%), and C30 (61.2% and 95.3%); and fatty acids C20 (79.8% and 23.0%), C22 (162.0% and 176.0%), C24 (75.9% and 60.6%), and C26 (140.9% and 200.8%) were detected under the water deficit and 20% PEG6000 treatments, respectively (**Figure 1B**, **Supplementary Table 2**). These data showed that alkanes were more sensitive to drought stress than other wax compounds, implying that alkanes biosynthesis may play an important role in drought resistance of *B. distachyon*.

### Identification of *CER1* Homologous Genes in *B. distachyon*

**Figure 2:**
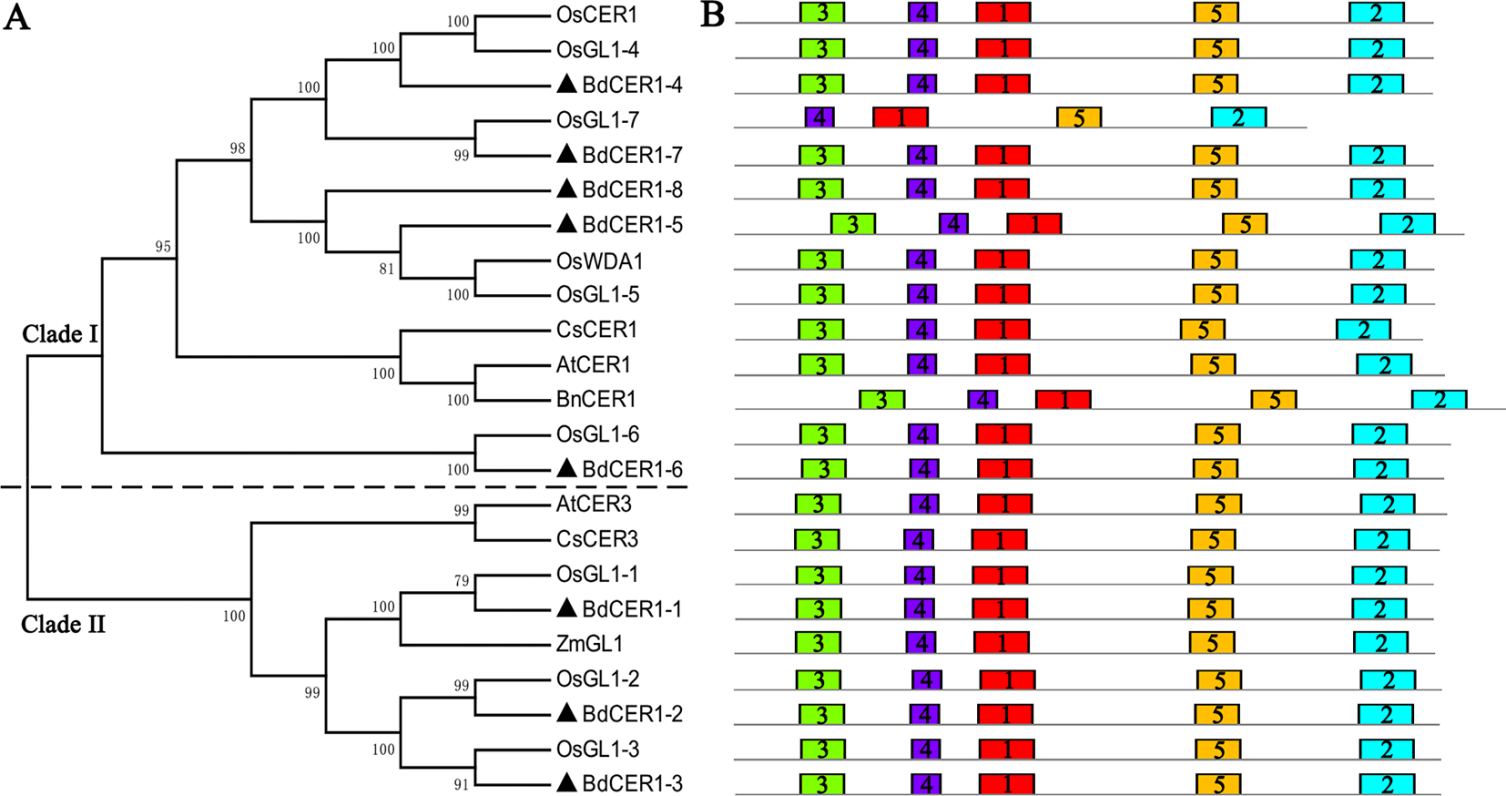
Phylogenetic and protein structure analyses on homologous genes of *CER1* in various plants. **(A)** Phylogenetic tree. Black triangles point to candidate genes in this study. Two significant clades separately belong to homologous genes of *CER1* (Clade I) and *CER3* (Clade II). The abbreviations of diverse species: At, *Arabidopsis thaliana*; Bn, *Brassica napus*; Cs, *Cucumis sativus*; Zm, *Zea mays*; Os, *Oryza sativa*; Bd, *Brachypodium distachyon*. **(B)** Five putative motifs in *CER1* homologous genes identified by MEME. Different color rectangles indicate putative motifs

The Arabidopsis CER1 protein was used to search against *B. distachyon* genome, and eight putative *CER1* homologous genes located on four different chromosomes were found, which were separately named as *BdCER1-1* to *BdCER1-8* (**Table 1**). Although the number of exons varied from 8 to 10, there were no significant differences in coding DNA sequences (CDS), amino acid lengths, pIs, and MWs (**Table 1**). To determine evolutionary relationships among the *BdCER1s*, their amino acid sequences together with other 15 reported orthologous CER1s were used to construct a phylogenetic tree. Based on the phylogenetic tree topology, all proteins were characterized into two main clades (I and II), of which clade I and clade II mainly contained the CER1-related and the CER3-related members, respectively (**Figure 2A**), suggesting that function diversification between *CER1* and *CER3* seemed to have taken place before monocot/dicot separation. As a result, *CER1* and *CER3* might represent most of their individual ancestor functions, respectively. In *B. distachyon, BdCER1-4*, *-5*, *-6*, *-7*, and *-8* were classified into clade I, while *BdCER1-1*, *-2*, and *-3* were clustered into clade II (**Figure 2A**), implying that the duplication events of *BdCER1s* happened after the separation of dicot from monocot, indicating that the formation of *BdCER1s* was independent and their functions might also be diverse to some extent.

**Table 1:** *CER1* homologous genes in *Brachypodium distachyon*.

Gene symbol	Protein name	Exons	CDS (bp)	Length aa)	MW (kDa)	pI
*BdCER1-1*	Bradi4g30750.1	9	1859	619	69.82	9.46
*BdCER1-2*	Bradi3g05880.1	10	1880	626	70.74	9.41
*BdCER1-3*	Bradi1g31140.1	8	1859	626	70.78	9.33
*BdCER1-4*	Bradi3g48997.1	10	1860	619	71.28	8.93
*BdCER1-5*	Bradi3g28460.1	9	1944	647	73.90	9.05
*BdCER1-6*	Bradi3g55100.1	9	1890	629	70.47	8.35
*BdCER1-7*	Bradi5g15480.6	10	1863	620	71.43	9.18
*BdCER1-8*	Bradi3g28450.1	10	1865	621	71.04	8.90

By using the MEME program, five putative motifs were identified and annotated in BdCER1s protein sequences (**Figure 2B**, **Supplementary Figure 1**). In the FAH superfamily, three His-rich motifs (H-X3-H, H-X2-HH, and H-X2-HH, where X represents any amino acid) were present in all BdCER1s members (**Supplementary Figure 2**), which are very important for *CER1* and *CER1-like1* genes to function in VLC alkanes biosynthesis (Bourdenx et al., 2011; Pascal et al., 2019). These analyses suggested that *BdCER1*s may be candidate genes involved in VLC alkanes biosynthesis in *B. distachyon*.

### Expression Profiles of *BdCER1* Genes in Various Tissues and Under Different Stresses

**Figure 3:**
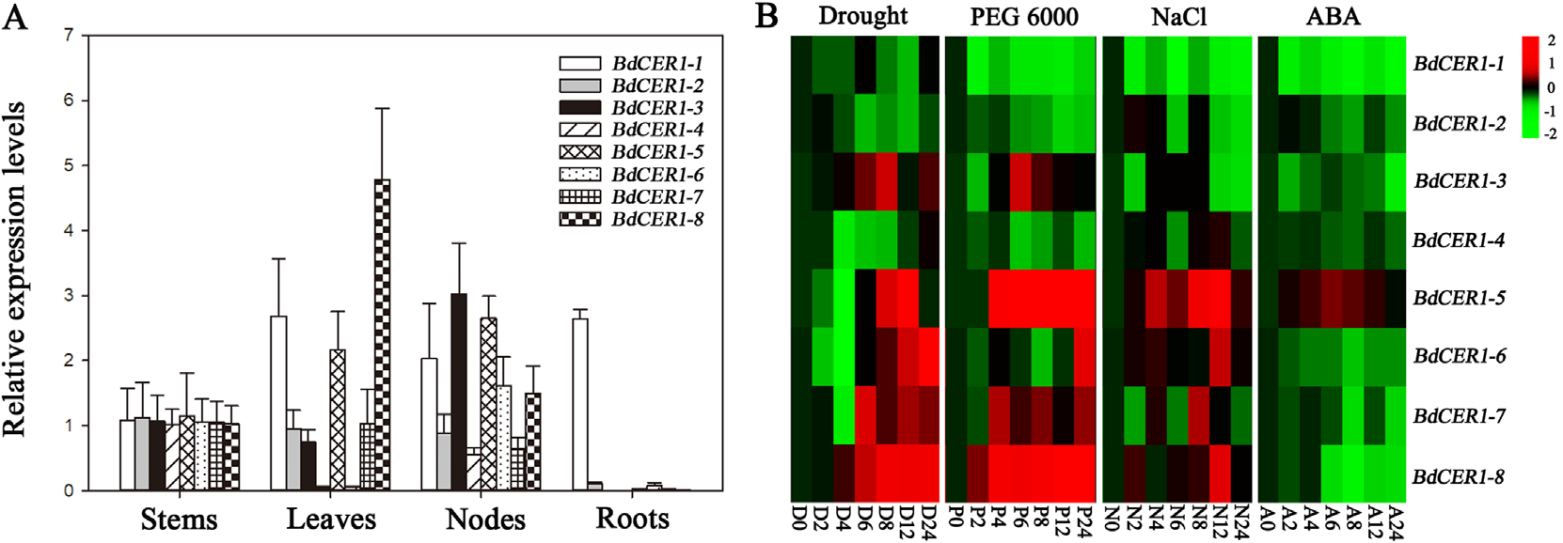
Gene expression analysis of *BdCER1s*. **(A)** Tissue expression analysis. **(B)** Expression analysis under drought (D), PEG (P), ABA (A), and NaCl (N) treatments. Results are presented as relative expression levels. The data represent the means ± standard deviations of three biological replicates.

The expression levels of *BdCER1* genes in four tissues of Bd21 including leaves, stems, nodes, and roots were examined by qRT-PCR (**Figure 3A**). *BdCER1-1* exhibited relatively high expressions in all tissues. The expression of *BdCER1-2* and *-7* were detected in all tissues except in roots. The high expression levels of *BdCER1-3*, *-4*, and *-6* were detected in stems and nodes. Relatively high expression was detected in stems, leaves, and nodes for *BdCER1-5*. Interestingly, *BdCER1-8* was preferentially expressed in leaves and its expression level was the highest among all *BdCER1* genes examined, which was in agreement with the electronic expression levels of *BdCER1* genes in leaves according to the Plant Genomics Resource database (**Supplementary Figure 3**). These analyses showed that *BdCER1* genes may be involved in wax accumulation of different tissues.

To investigate the roles of *BdCER1* genes in stress resistance, the transcript levels of *BdCER1* genes under drought, PEG6000, ABA, and NaCl treatments were analyzed using qRT-PCR (**Figure 3B**). Among them, expressions levels of five genes (*BdCER1-3*, *-5*, *-6*, *-7*, and *-8*) under drought and PEG6000 treatments were gradually increased throughout the time course. However, expressions of *BdCER1-5*, *-6*, *-7*, and *-8* with NaCl treatment showed diversified patterns. The expression level of *BdCER1-5* was constitutively increased throughout the time course. The expression levels of *BdCER1-6* and *-8* peaked at 12 h after the treatment and then became decreased, whereas the *BdCER1-7* only showed strong induction at 8 h after the treatment. Expression of only one gene (*BdCER1-5*) was induced by ABA treatment, and its expression level was gradually increased within 12 h after the treatment and then gradually went decreased. These analyses indicated that *BdCER1* genes may participate in stress responses and they may play diverse roles in different stress pathways.

### Molecular and Phenotypic Characterizations of *BdCER1-8* Transgenic Plants

**Figure 4:**
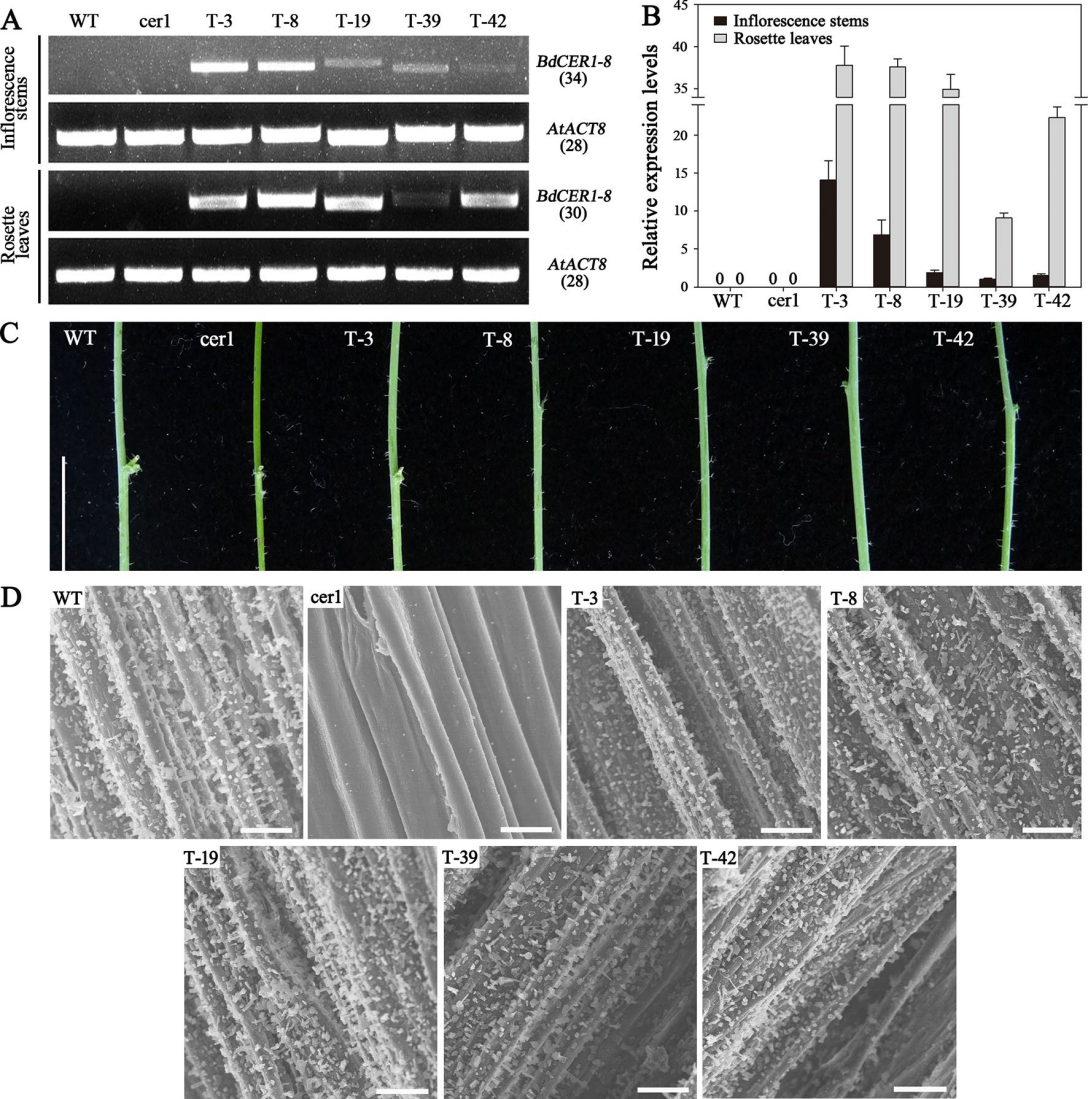
Molecular and phenotypic characterizations of *BdCER1-8* transgenic plants. **(A)** Semi-quantitative RT-PCR analysis of *BdCER1-8* transcripts in WT plant, the *cer1* mutant, and the transgenic lines with *BdCER1-8* transgene. **(B)** qRT-PCR analysis of *BdCER1-8* gene expression in the WT plant, the *cer1* mutant, and the transgenic lines with *BdCER1-8* transgene. The *AtACT8* gene is used as a constitutively expressed control. Results are presented as relative expression levels of T-39 transgenic line in stem. The data represent the means ± standard deviations of three technical replicates. **(C)** Stem color comparison among WT, *cer1*, and transgenic plants. Bars = 1 cm. **(D)** Epicuticular wax crystal of stems from WT, *cer1*, and transgenic plants observed by SEM at 1,000 × magnification. Bars = 10 μm

Due to its unique features including high expression level in leaf where wax synthesis is active and intense responses to osmotic stresses, *BdCER1-8* was selected as representation of *BdCER1* family genes for functional study by introducing its full-length CDS into Arabidopsis *cer1* mutant. Eleven T3 independent transgenic lines were used to detect a 3:1 separation ratio. Among these, five homozygous transgenic lines were obtained and further analyzed. Semi-quantitative RT-PCR showed that the *BdCER1-8* expression was detected in all five transgenic lines but not detected in the *cer1* mutant and WT plant (**Figure 4A**), which was further confirmed by qRT-PCR analysis (**Figure 4B**). The Arabidopsis *CER1* gene was expressed at very low levels in both the *cer1* mutant and the transgenic lines compared to that in WT plant. Whereas the *BdCER1-8* transgene was extremely highly expressed in transgenic lines and was absent in the WT and *cer1* plants (**Figure 4B**, **Supplementary Figure 4**), indicating that *BdCER1-8* gene had been successfully expressed in the *cer1* mutant without interfering the normal remain of *CER1* function in the *cer1* mutant.

To examine the biological function of *BdCER1-8*, its heterologous expression in the *cer1* mutant could be reflected by viewing the stem color (**Figure 4C**). Compared with WT plant, the stem surface of the *cer1* mutant exhibited a glossy green phenotype. However, the *BdCER1-8* heterologous expression in the *cer1* mutant could prevent this glossy green phenotype and displayed a similar glaucous color to the WT plant. To confirm these observations in details, the stem surfaces of different lines were observed by SEM (**Figure 4D**). Indeed, compared with the WT plant, the stem surface of the *cer1* mutant did not have wax crystal formation, whereas the *BdCER1-8* heterologous expression in the *cer1* mutant restored its wax crystal formation, suggesting that the stem wax formation defect in the *cer1* mutant could be rescued by *BdCER1-8*.

### *BdCER1-8* Overexpression Rescues Wax Biosynthesis in the Arabidopsis *cer1* Mutant

**Figure 5:**
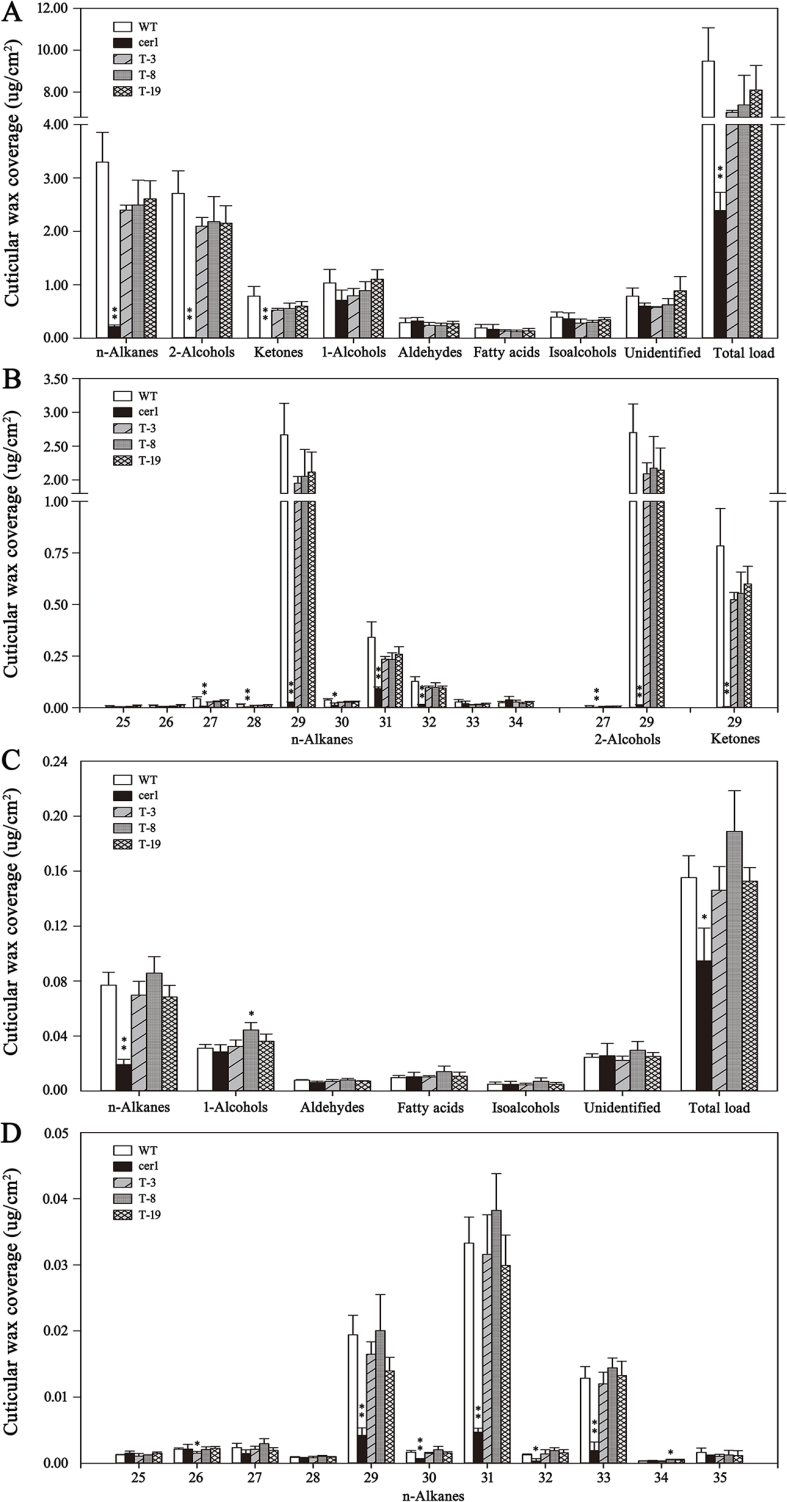
Cuticular wax amount and composition in inflorescence stems and rosette leaves of WT, *cer1*, and transgenic plants. **(A** and **B)** are cuticular wax loads and compositions in the inflorescence stems of different lines, respectively. **(C** and **D)** are cuticular wax loads and compositions in the rosette leaves of different lines, respectively. The results show averages of three biological replicates, and error bars indicate ± SD. Asterisks indicate significant differences from WT plant (t-test: * for p < 0.05; ** for p < 0.01). Numbers on the x-axis of **(B** and **D)** indicate carbon numbers of the compounds.

Cuticular wax accumulation on stems and leaves from different lines including the WT plant, the *cer1* mutant, and three transgenic lines with relatively high *BdCER1-8* expressions (T-3, T-8, and T-19) was analyzed in details (**Figure 5**). Compared with the *cer1* mutant, the total wax contents on stems was increased by 66.1%, 67.7%, and 70.5% in T-3, T-8, and T-19 lines, respectively. This change was largely due to an increased content of alkanes, ketones, and secondary alcohols that are produced through the alkane-forming pathway (**Figure 5A**, **Supplementary Table 3**). Further, the most prominent changes were contributed by C29 alkane, C29 ketone, and C29 secondary alcohol (98.9%, 99.4%, and 99.5% increases, respectively) (**Figure 5B**, **Supplementary Table 4**). On the contrary, the contents of other wax components were very slightly changed (**Figure 5A**, **Supplementary Table 3**). The heterologous expression of *BdCER1-8* restored stem wax content of the *cer1* mutant to 74.2–85.5% of that of the WT plant, but the stem total wax contents between transgenic lines and WT plant did not reach significant differences (**Figure 5A**, **Supplementary Table 3**), suggesting that *BdCER1-8* alone might be sufficient for the restoration of wax loss in the *cer1* stem.

In leaves, the total wax contents of T-3, T-8, and T-19 lines were also significantly increased compared with those in the *cer1* mutant (35.2%, 49.9%, and 38.0% increases, respectively), which was due to the increases of alkane amounts (around 74.0% increases) (**Figure 5C**, **Supplementary Table 5**). More precisely, this modification was largely contributed by the increases of the C29, C31, and C33 alkane contents (74.5%, 85.7%, and 85.4% increases, respectively) (**Figure 5D**, **Supplementary Table 6**). Nevertheless, there were only slight changes for other wax components (**Figure 5C**, **Supplementary Table 5**). The heterologous expression of *BdCER1-8* in the *cer1* mutant restored leaf total wax content to 91.1–117.9% of that in WT plant (**Figure 5C**, **Supplementary Table 5**). All these results suggested that *BdCER1-8* rescued wax production through its participation in the alkane-forming pathway in the transgenic lines.

### *BdCER1-8* Overexpression Changes Cuticle Properties and Susceptibility to Soil Water Deficit of the *cer1* Mutant

**Figure 6:**
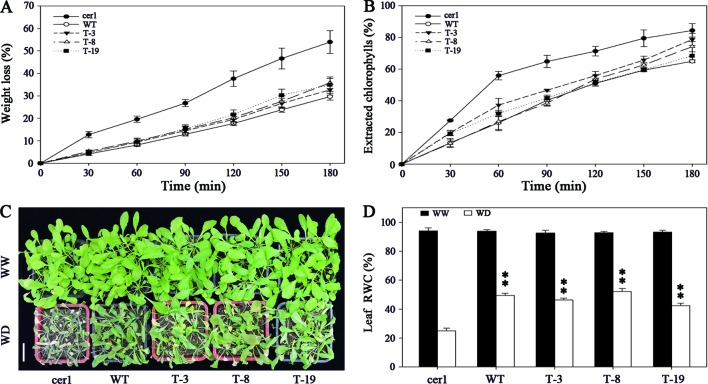
*BdCER1-8* alters cuticle properties and response to soil water deficit of the *cer1* mutant. **(A)** Water-loss rates of isolated rosette leaves from WT, *cer1*, and transgenic plants. **(B)** Chlorophyll leaching rates of isolated rosette leaves from different lines as indicated above. **(C)** Soil water deprivation experiment. The abbreviations of WW and WD indicate well-watered plants and water-deprived plants, respectively. Bar = 3 cm. **(D)** Leaf RWC of well-watered and water-deprived plants. Data are shown by mean ± SD of four biological replicates. Asterisks indicate significant differences from the *cer1* mutant (t-test: * for p < 0.05; ** for p < 0.01).

Many studies have indicated that change in wax content is generally related to altered cuticle permeability of plants (Kosma et al., 2009; Bourdenx et al., 2011; Xu et al., 2014; Wang et al., 2015a, Wang et al., 2015b; Zhou et al., 2015; Ni et al., 2018). Thus, we conducted chlorophyll leaching and water loss assays in WT plant, *cer1* mutant, and T-3, T-8, and T-19 transgenic lines to assess whether the impact of *BdCER1-8* over-expression would affect cuticle permeability of the *cer1* mutant (**Figure 6A, B**). The rates of both water loss and chlorophyll leaching in the transgenic lines were lower than those in the *cer1* mutant but similar to those in the WT plant at all examined times, indicating that the *BdCER1-8* heterologous expression changed cuticle properties of the *cer1* mutant. To further test responses of the transgenic lines to soil water deficit, 4-week-old lines were not watered until the *cer1* mutant became wilted (**Figure 6C**). After 10 days of water deprivation treatment, the wilted *cer1* mutant showed a relative water content (RWC) of 25.1%. In contrast, transgenic plants showed a less sensitive response. Compared with the *cer1* mutant, the transgenic lines maintained a high RWC of 46.3%, 52.1%, and 42.4% in T-3, T-8, and T-19 lines, respectively (**Figure 6D**). These data suggested that cuticle changes induced by *BdCER1-8* decreased the susceptibility of the *cer1* mutant to soil water deficit.

## Discussion

In this study, we systematically investigated the influence of drought stress on the leaf cuticular wax accumulation in *B. distachyon*. Our data indicate that alkanes were more sensitive to drought stress than other wax compounds. Then, we identified eight putative homologous genes of *CER1* involved in the VLC alkane biosynthesis of *B. distachyon*, and further suggested that *BdCER1-8* was predominant in VLC alkanes production of leaf due to its relatively high expression levels in leaf and its capability to have nearly fully rescued Arabidopsis *cer1* mutant phenotype.

The accumulation of leaf cuticular wax is an important physiological process in plants, which is also seriously affected by environmental stresses (Jenks et al., 2002). Drought stress often results in significant increase of cuticular wax in many plants (Cameron and Smart, 2006; Kosma et al., 2009; Xu et al., 2014; Xu et al., 2016). In Arabidopsis, the leaf wax content was increased by 75% after drought stress, primarily due to increases in wax alkanes (Kosma et al., 2009). Additionally, the content of alkanes was also the most significant change in the Yukon ecotype of saltwater cress and *Populus euphratica* after water deficit (Xu et al., 2014; Xu et al., 2016). Above results imply that the synthesis of alkanes plays a key role in drought stress response. In our study, to assess the influence of drought stress on leaf cuticular wax accumulation in *B. distachyon*, both water deficit and PEG treatments were performed. Results showed that the cuticular wax of control plants mainly contained primary alcohols, alkanes, aldehydes, and fatty acids, in which primary alcohols were dominant components with few contents of other compounds (**Figure 1**), which is consistent with the results of a recent study that the leaf cuticular wax of *B. distachyon* mainly consists of primary alcohols with less contents of aldehydes, alkanes, and fatty acids throughout leaf development (Wang et al., 2018). All treatments resulted in significant increases of total wax load and individual compound without changing wax composition (**Figure 1**). However, the change of alkanes content was mostly obvious among all wax compounds, suggesting that alkanes biosynthesis may play an indispensable role in drought resistance of *B. distachyon*.

The Arabidopsis *CER1* gene encodes an essential enzyme involved in the VLC alkane biosynthesis with the presence of FAH superfamily and WAX2 C-terminal domain (Bourdenx et al., 2011; Bernard et al., 2012). The FAH superfamily is functionally involved in zeaxanthin synthesis, cholesterol biosynthesis, and plant cuticular wax biosynthesis (Arthington et al., 1991; Aarts et al., 1995; Bard et al., 1996), in which three His-rich motifs are essential for *CER1* and *CER1-like1* to function in VLC alkane biosynthesis (Bernard et al., 2012; Pascal et al.,2019). By analyzing FAH superfamily of CER1-related proteins from monocots and dicots (**Supplementary Figure 2**), we observed that the three His-rich motif core is a shared common feature, indicating that homologous genes of *CER1* could be functionally similar and are likely evolved from the same ancestor with His-rich motifs. But according to the phylogenetic tree, they were separately characterized into the CER1-related and the CER3-related groups, respectively (**Figure 2A**), suggesting that their functional diversification seems to have taken place before monocot/dicot separation, causing subsequent gains of new functions during the long evolutionary history. Additionally in rice, the disruptions in functions of *OsGL1*s including *OsGL1-1*, *OsGL1-2*, *OsGL1-3*, and *OsGL1-6* affect cuticular wax biosynthesis and drought resistance capability in leaf, whereas others including *OsGL1-4/OsCER1* and *OsGL1-5/WDA1* are involved in VLC alkanes biosynthesis and pollen development in anther (Jung et al., 2006; Islam et al., 2009; Qin et al., 2011; Zhou et al., 2013; Zhou et al., 2015; Ni et al., 2018). These *OsGL1*s were phylogenetically more closely related to *BdCER1*s, and their corresponding orthologs were also identified to be the *BdCER1* family genes (**Figure 2A**), indicating that though *BdCER1*s have multiple copies (five *CER1*-like genes and three *CER3*-like genes) and shared core motifs (H-X3-H, H-X2-HH, and H-X2-HH, where X represents any amino acid), they have experienced extensive neofunctionalization since their duplication events because their independent gene birth allow diversification during this long history.

The neofunctionalization consequences may also be related with the differential expression profiles of *BdCER1* genes in various tissues (**Figure 3A**), which is similar to what their rice homologous genes (*OsGL1*s) behave in different organs to affect wax accumulation in leaf or anther (Jung et al., 2006; Islam et al., 2009; Qin et al., 2011; Zhou et al., 2013; Zhou et al., 2015; Ni et al., 2018). Thus, in consideration of the tissue expression profiles of *BdCER1* family genes (**Figure 3A**), we further inferred that *BdCER1-1*, *-3*, and *-5* might play key roles in cuticular wax biosynthesis of node where *BdCER1-1* might also play an important role in wax production of root. In contrast, *BdCER1-8* mainly affected cuticular wax accumulation in leaf. In Arabidopsis, although *CER1* and *CER1-like1* show different tissues expression levels, they separately interact with *CER3* and *CYTB5-B* to produce VLC alkanes in yeast (Bourdenx et al., 2011; Bernard et al., 2012; Pascal et al., 2019). As such, we provide a model that *CER1*-related genes (*BdCER1-4*, *-5*, *-6*, *-7*, and *-8*) maybe separately form alkane-forming complexes with *CER3*-related genes (*BdCER1-1*, *-2*, and *-3*) to participate in VLC alkanes accumulation of different tissues; while as specific cofactors of *CER1-*related genes, *CYTB5-B* family genes function to improve their efficiencies in this process. This model needs further bench work confirmation in the future study.

Heterologous expression of *BdCER1-8* in the Arabidopsis *cer1* mutant mainly resulted in increased contents of alkanes and their derivatives in leaf or stem, whereas other wax compounds were only slightly changed (**Figure 5**), indicating that *BdCER1-8* is involved in the alkane-forming pathway in the *cer1* mutant. Even though *B. distachyon* genome encodes multiple CER1-homologous proteins, our analysis suggests that *BdCER1-8* may play a more predominant role in alkane production of leaf than its paralogs because it has the highest expression level in leaf among all *BdCER1*s and it along can nearly completely restored the *cer1* defects. This explicit and dominant function of *BdCER1-8* may suggest extensive functional diversification existing among *BdCER1*s, allowing optimized spatial and temporal regulation of wax synthesis in different tissues.

On the other hand, three *BdFAR* genes (*BdFAR1*, *BdFAR2*, and *BdFAR3*) have been demonstrated to be involved in the alcohol-forming pathway and they respond to abiotic stresses in *B. distachyon* (Wang et al., 2018). These results together suggest that the cuticular wax biosynthetic pathways in *B. distachyon* are similar to those in Arabidopsis, containing both the alcohol-forming and the alkane-forming pathways. The leaf cuticular wax of *B. distachyon* consists of primary alcohols, aldehydes, alkanes, and fatty acids, among which primary alcohols are the dominant wax components throughout leaf development, accounting for 82–89% of total wax contents (Wang et al., 2018). Our work also shows that primary alcohols are the dominant wax components, which accounts for 89.6% of total wax load in control plants (**Figure 1A**, **Supplementary Table 1**). These results further suggest that the alcohol-forming pathway is a dominant wax-forming pathway in *B. distachyon*. VLCFAs are common precursors for both the alcohol-forming and the alkane-forming pathways (Samuels et al., 2008; Kunst and Samuels, 2009). Thus, we propose that *BdFARs* may be predominant in competing VLCFAs from *BdCER1s*, which results in producing high level of primary alcohols in *B. distachyon* leaf.

The biological impact of *CER1* genes can be interpreted by the water loss rate and chlorophyll leaching rate tests on detached leaves (**Figures 5A, B**), where water loss rates and chlorophyll leaching rates in transgenic lines showed lower levels than those in the *cer1* mutant, but were similar to those in the WT plant at all examined time. Additionally, transgenic plants contained a relatively higher water content than the *cer1* mutant after 10 days of water deprivation treatment (**Figures 5C, D**). All data imply that *BdCER1-8* overexpression changes the repelling properties of water loss in the leaf cuticle of the *cer1* mutant, which can be caused by the increased alkanes content. Similar effect was also observed in other species. In cucumber, the *CsWAX2* overexpression reduced cuticle permeability of the *wax2* mutant by rescuing the alkane-forming pathway (Wang et al., 2015a). Further, abnormal expressions of *CsCER1* and *CsWAX2* in transgenic cucumber plants have significant effects on VLC alkanes biosynthesis, which finally changes resistance to water loss in the transgenic plants from that in the wild type (Wang et al., 2015a, Wang et al., 2015b). These results further supports our conclusion that alkanes biosynthesis plays a crucial role in drought resistance of *B. distachyon*.

It is also generally known that wax-related genes are involved in regulatory networks of cuticular wax biosynthesis under diverse environments (Lee and Suh, 2013). Many genes, such as *CER1* in Arabidopsis (Kosma et al., 2009), *OsGL1s* in rice (Jung et al., 2006; Islam et al., 2009; Qin et al., 2011; Zhou et al., 2013; Zhou et al., 2015), *CsCER1* and *CsWAX2* in cucumber (Wang et al., 2015a, Wang et al., 2015b), and *BdFARs* in *B. distachyon* (Wang et al., 2018), respond to drought, sodium chloride, cold, and ABA treatments. In our study, qRT-PCR was performed to elucidate the expression patterns of *BdCER1* genes under ABA, drought, PEG, and sodium chloride treatments. The results revealed that *BdCER1* genes exhibited different responses to these abiotic stresses. Under water deficit and PEG treatments, *BdCER1-3*, *-5*, *-6*, *-7*, and *-8* were significantly up-regulated (**Figure 3B**), which could explain the obvious alkane induction on *B. distachyon*, and perhaps provide a drought resistance mechanism for reducing cuticle permeability by increasing wax accumulation. But only *BdCER1-5* were significantly up-regulated under ABA treatment (**Figure 3B**), suggesting that they may be involved in different regulatory networks of wax accumulation under drought stress. In Arabidopsis, the transcript levels of *MYB94* or *MYB96* are significantly up-regulated in response to ABA-mediated drought signals (Seo et al., 2011; Lee and Suh 2015). Their proteins directly bind to the gene promoters to activate cuticular wax biosynthetic genes, which finally results in cuticular wax accumulation (Seo et al., 2011; Lee and Suh 2015). As a homolog of *MYB96* in wheat, *TaMYB31* is also a drought-induced transcription factor involved in cuticle biosynthetic pathway (Bi et al., 2016). Therefore, it can be speculated that *BdCER1-5* might be involved in ABA-mediated transcription factors activation in *B. distachyon* to stimulate wax biosynthesis in response to drought stress, possibly through interacting with homologs of *MYB94* or *MYB96*. In contrast, although *BdCER1-3*, *-6*, *-7*, and *-8* were significantly up-regulated by drought and PEG treatments, they were down-regulated under ABA treatment (**Figure 3B**), suggesting that these genes may be involved in other regulatory network to cope with drought stress. Thus, the mechanism how *BdCER1-3*, *-6*, *-7*, and *-8* function in this process and their interacting partners remain to be elucidated.

## Data Availability Statement

The raw data supporting the conclusions of this manuscript will be made available by the authors, without undue reservation, to any qualified researcher.

## Author Contributions

HW, YC, and LQ contributed to the experimental design. HW, SS, XL, TinL, JW, TiaL, QZ, WS, CL and ZW performed the experiments and analyzed the data. HW and LQ wrote the manuscript and YC revised it. All authors were involved in the revision of the manuscript and approved the final manuscript.

## Funding

This research was supported by the Science and Technology Innovation Team Project (2014KCT-25), National Natural Science Foundation of China (31500256), the Key Research and Development Project of Shaanxi Province (No. 2019ZDLNY04-05), and Tobacco Company Project (No. KJ-2016-01) of Shaanxi Province, China.

## Conflict of Interest

The authors declare that the research was conducted in the absence of any commercial or financial relationships that could be construed as a potential conflict of interest.

## Abbreviations

VLC, very-long-chain; VLCFA, very-long-chain fatty acid; FAH, fatty acid hydroxylase; PEG, polyethylene glycol; CYTB5s, cytochrome b5 isoforms; GC-MS, gas chromatography-mass spectrometry; GC-FID, gas chromatography with flame ionization detector; qRT-PCR, quantitative real-time PCR; RWC, relative water content; SEM, scanning electron microscopy; CDS, coding DNA sequence; bp, base pair; aa, amino acid; MW, molecular weight; kDa, kilodalton; pI, isoelectric point.
