# Supplementary material for: Expression Analysis and Functional Characterization of CER1 Family Genes Involved in Very-Long-Chain Alkanes Biosynthesis in Brachypodium distachyon
Source: Front Plant Sci. 2019 Nov 1;10:1389. doi: 10.3389/fpls.2019.01389 (PMC6838206; doi:10.3389/fpls.2019.01389)
Supplement: Supplementary file 1 [file Presentation_1.pptx]

## Slide 1
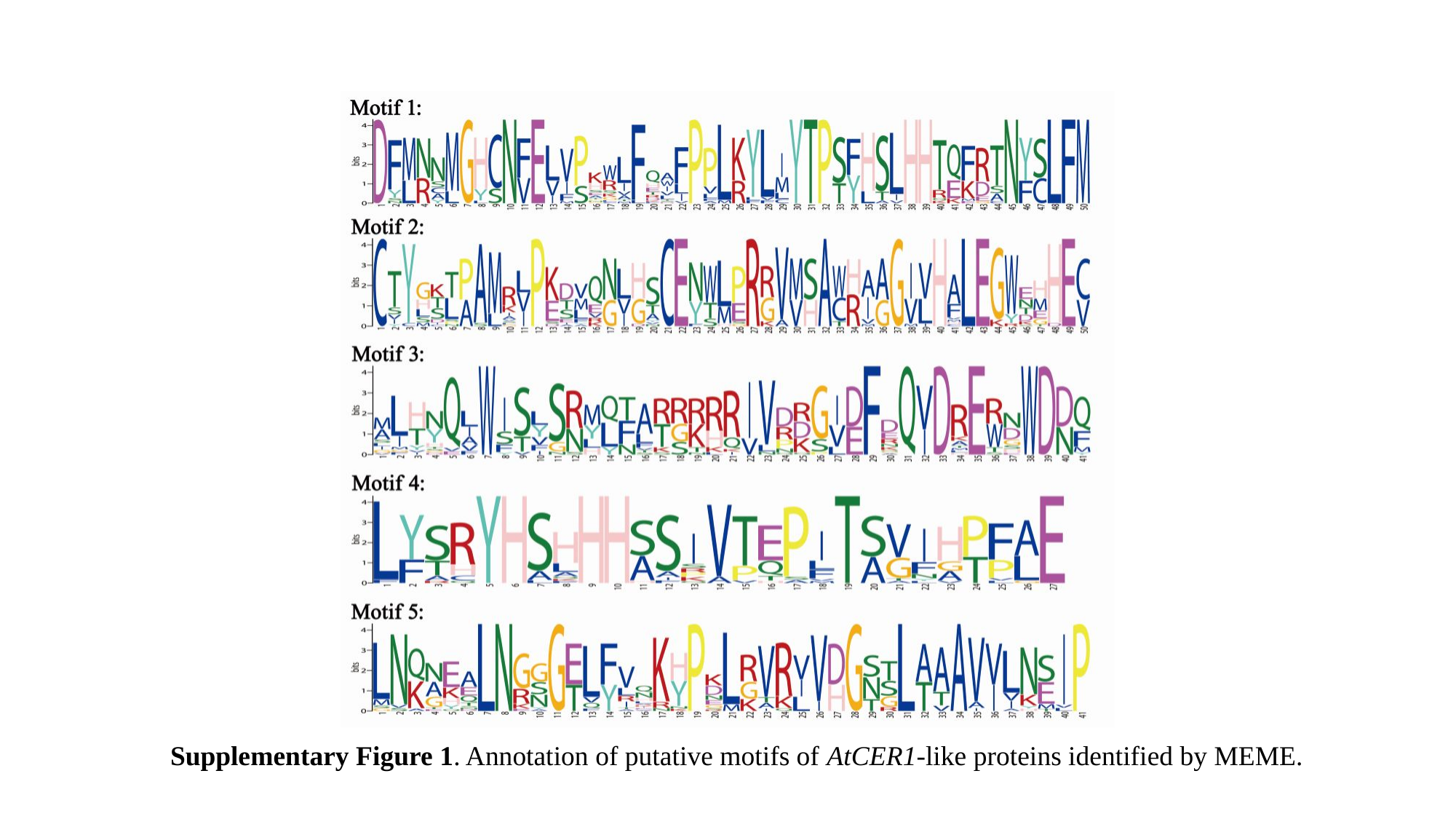

Supplementary Figure 1. Annotation of putative motifs of AtCER1-like proteins identified by MEME.
