# Supplementary material for: Expression Analysis and Functional Characterization of CER1 Family Genes Involved in Very-Long-Chain Alkanes Biosynthesis in Brachypodium distachyon
Source: Front Plant Sci. 2019 Nov 1;10:1389. doi: 10.3389/fpls.2019.01389 (PMC6838206; doi:10.3389/fpls.2019.01389)
Supplement: Supplementary file 2 [file Presentation_2.pptx]

## Slide 1
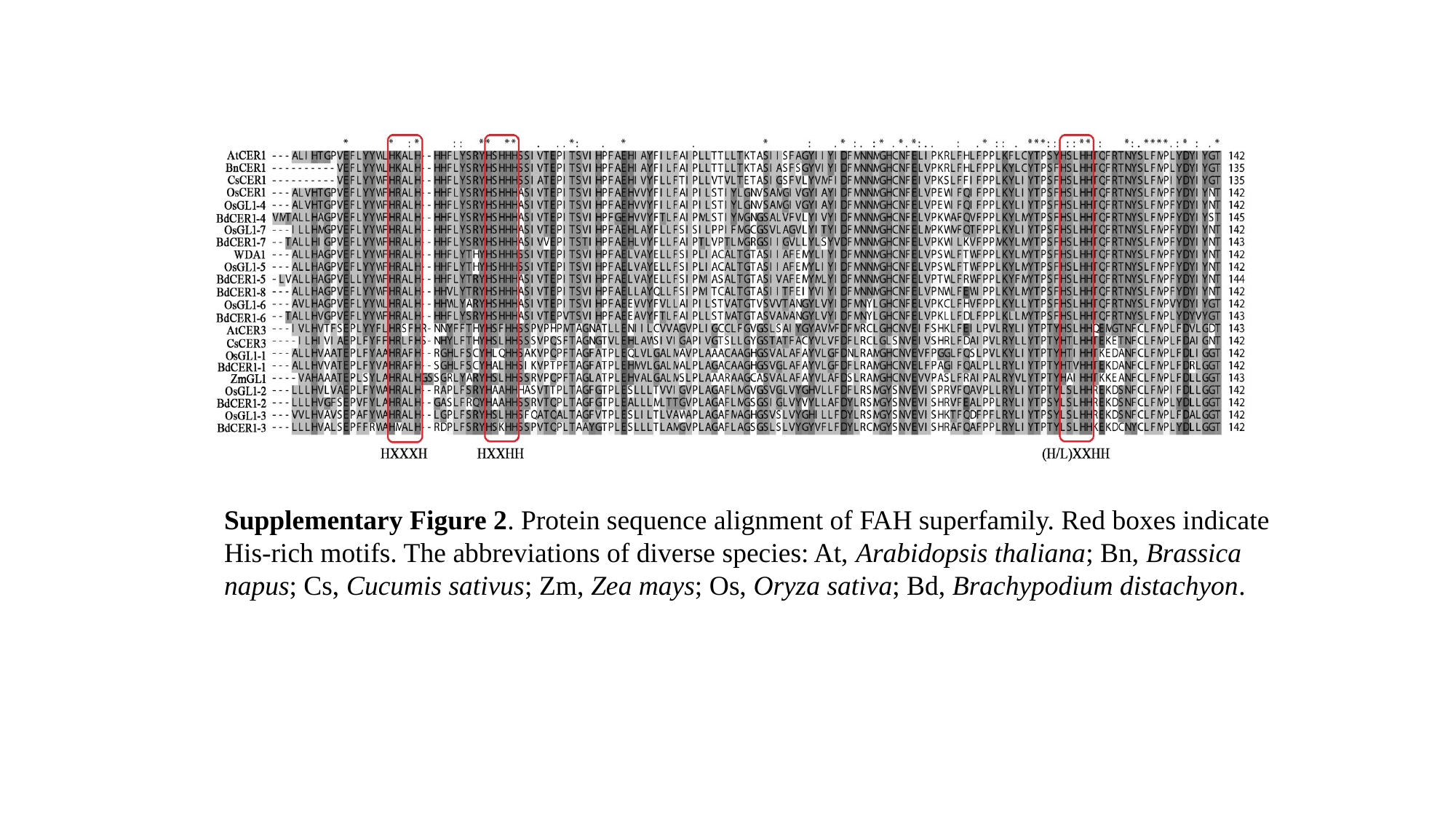

Supplementary Figure 2. Protein sequence alignment of FAH superfamily. Red boxes indicate His-rich motifs. The abbreviations of diverse species: At, Arabidopsis thaliana; Bn, Brassica napus; Cs, Cucumis sativus; Zm, Zea mays; Os, Oryza sativa; Bd, Brachypodium distachyon.
