# Supplementary material for: Expression Analysis and Functional Characterization of CER1 Family Genes Involved in Very-Long-Chain Alkanes Biosynthesis in Brachypodium distachyon
Source: Front Plant Sci. 2019 Nov 1;10:1389. doi: 10.3389/fpls.2019.01389 (PMC6838206; doi:10.3389/fpls.2019.01389)
Supplement: Supplementary file 4 [file Presentation_4.pptx]

## Slide 1
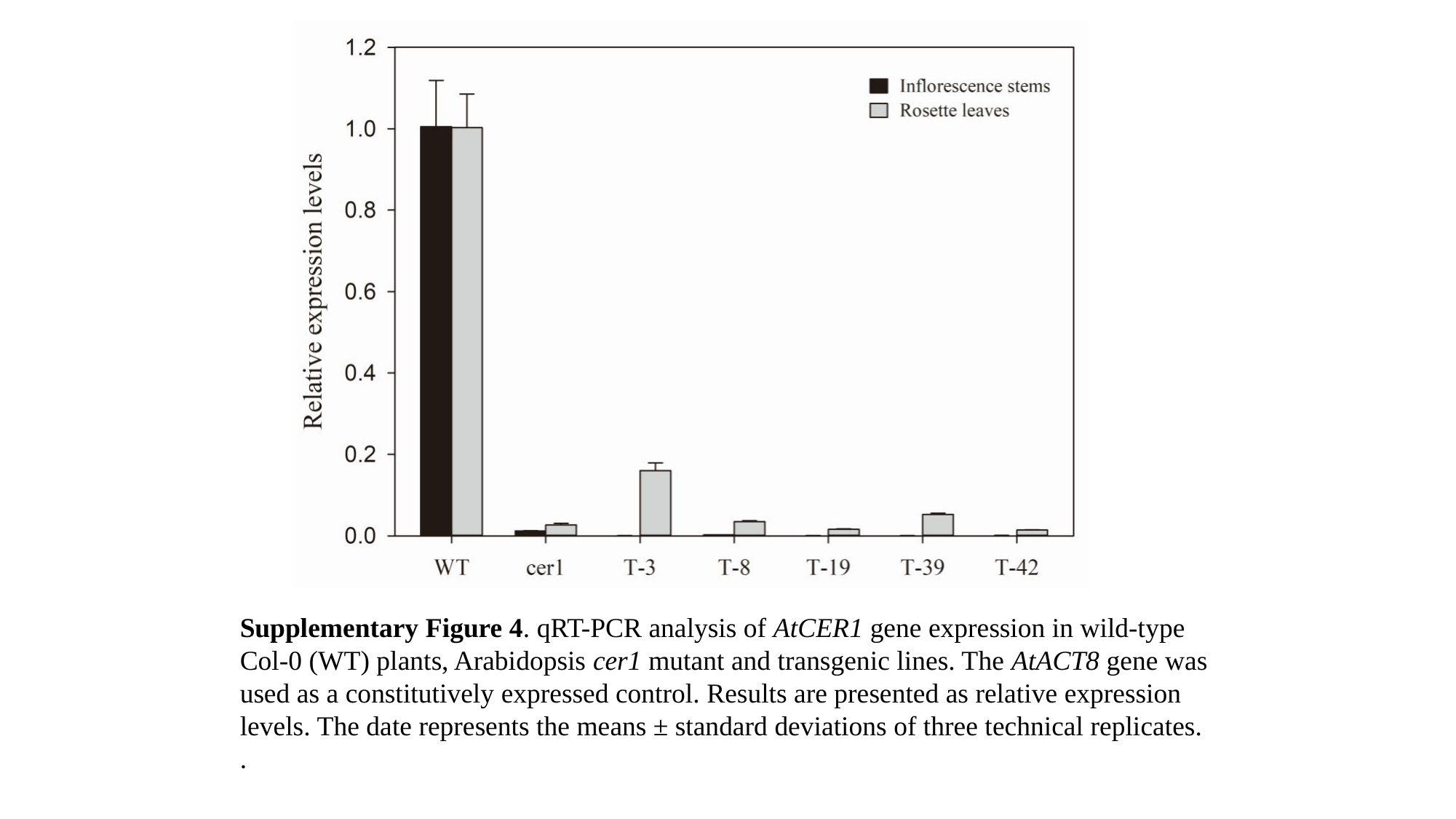

Supplementary Figure 4. qRT-PCR analysis of AtCER1 gene expression in wild-type Col-0 (WT) plants, Arabidopsis cer1 mutant and transgenic lines. The AtACT8 gene was used as a constitutively expressed control. Results are presented as relative expression levels. The date represents the means ± standard deviations of three technical replicates.
.
