# Supplementary material for: Expression Analysis and Functional Characterization of CER1 Family Genes Involved in Very-Long-Chain Alkanes Biosynthesis in Brachypodium distachyon
Source: Front Plant Sci. 2019 Nov 1;10:1389. doi: 10.3389/fpls.2019.01389 (PMC6838206; doi:10.3389/fpls.2019.01389)
Supplement: Supplementary file 5 [file Table_1.doc]

| **Supplementary Table 1**. Wax constituents of *Brachypodium distachyon* ‘Bd21’ leaves | | | | | |
| --- | --- | --- | --- | --- | --- |
| Wax contents | Control (μg/cm^2^) |  | Water deficit (μg/cm^2^) |  | 20% PEG6000 (μg/cm^2^) |
|  | Mean ± SD |  | Mean ± SD |  | Mean ± SD |
| Fatty acids | 0.140 ± 0.029 |  | 0.210 ± 0.052 |  | 0.210 ± 0.014 |
| Aldehydes | 0.126 ± 0.023 |  | 0.204 ± 0.038 |  | 0.176 ± 0.013 |
| Alkanes | 0.674 ± 0.046 |  | 1.408 ± 0.154 |  | 1.170 ± 0.266 |
| Primary alcohols | 8.103 ± 0.185 |  | 13.006 ± 0.746 |  | 13.013 ± 0.823 |
| Total load | 9.042 ± 0.160 |  | 14.828 ± 0.895 |  | 14.568 ± 1.075 |

| **Supplementary Table 2**. Individual wax constituents of *Brachypodium distachyon* ‘Bd21’ leaves | | | | | | |
| --- | --- | --- | --- | --- | --- | --- |
| Wax contents | Composition | Control (μg/cm^2^) |  | Water deficit (μg/cm^2^) |  | 20% PEG6000 (μg/cm^2^) |
|  |  | Mean ± SD |  | Mean ± SD |  | Mean ± SD |
| Fatty acids | C16:0 | 0.042 ± 0.014 |  | 0.052 ± 0.027 |  | 0.045 ± 0.013 |
|  | C18:0 | 0.024 ± 0.006 |  | 0.027 ± 0.011 |  | 0.026 ± 0.009 |
|  | C20:0 | 0.002 ± 0.000 |  | 0.004 ± 0.001 |  | 0.003 ± 0.000 |
|  | C22:0 | 0.003 ± 0.000 |  | 0.007 ± 0.002 |  | 0.007 ± 0.001 |
|  | C24:0 | 0.018 ± 0.002 |  | 0.031 ± 0.002 |  | 0.028 ± 0.002 |
|  | C26:0 | 0.022 ± 0.006 |  | 0.054 ± 0.004 |  | 0.067 ± 0.003 |
|  | C28:0 | 0.029 ± 0.004 |  | 0.035 ± 0.007 |  | 0.033 ± 0.008 |
|  |  |  |  |  |  |  |
| Aldehydes | C24 | 0.042 ± 0.004 |  | 0.071 ± 0.013 |  | 0.070 ± 0.004 |
|  | C26 | 0.016 ± 0.002 |  | 0.029 ± 0.005 |  | 0.026 ± 0.002 |
|  | C28 | 0.043 ± 0.018 |  | 0.063 ± 0.040 |  | 0.032 ± 0.010 |
|  | C30 | 0.025 ± 0.001 |  | 0.040 ± 0.007 |  | 0.049 ± 0.002 |
|  |  |  |  |  |  |  |
| Alkanes | C25 | 0.013 ± 0.003 |  | 0.024 ± 0.010 |  | 0.017 ± 0.003 |
|  | C27 | 0.026 ± 0.002 |  | 0.070 ± 0.007 |  | 0.055 ± 0.008 |
|  | C29 | 0.308 ± 0.015 |  | 0.773 ± 0.057 |  | 0.619 ± 0.171 |
|  | C31 | 0.299 ± 0.065 |  | 0.440 ± 0.116 |  | 0.399 ± 0.077 |
|  | C33 | 0.027 ± 0.002 |  | 0.100 ± 0.008 |  | 0.080 ± 0.015 |
|  |  |  |  |  |  |  |
| Primary alcohols | C22 | 0.013 ± 0.003 |  | 0.019 ± 0.005 |  | 0.014 ± 0.001 |
|  | C24 | 0.207 ± 0.076 |  | 0.671 ± 0.053 |  | 0.762 ± 0.042 |
|  | C26 | 7.654 ± 0.095 |  | 11.973 ± 0.682 |  | 11.916 ± 0.835 |
|  | C28 | 0.170 ± 0.005 |  | 0.237 ± 0.011 |  | 0.236 ± 0.008 |
|  | C30 | 0.033 ± 0.010 |  | 0.037 ± 0.010 |  | 0.034 ± 0.008 |
|  | C32 | 0.025 ± 0.021 |  | 0.068 ± 0.014 |  | 0.051 ± 0.011 |

| **Supplementary Table 3**. Cuticular wax composition of inflorescence stems of different Arabidopsis lines | | | | | | | | | |
| --- | --- | --- | --- | --- | --- | --- | --- | --- | --- |
| Wax | WT (μg/cm^2^) |  | cer1 (μg/cm^2^) |  | T-3 (μg/cm^2^) |  | T-8 (μg/cm^2^) |  | T-19 (μg/cm^2^) |
|  | Mean ± SD |  | Mean ± SD |  | Mean ± SD |  | Mean ± SD |  | Mean ± SD |
| Total load | 9.478 ± 1.588 |  | 2.388 ± 0.341 |  | 7.035 ± 0.094 |  | 7.389 ± 1.413 |  | 8.100 ± 1.172 |
| n-Alkanes | 3.297 ± 0.558 |  | 0.217 ± 0.028 |  | 2.396 ± 0.094 |  | 2.492 ± 0.467 |  | 2.607 ± 0.341 |
| Second alcohols | 2.709 ± 0.424 |  | 0.012 ± 0.003 |  | 2.096 ± 0.164 |  | 2.179 ± 0.472 |  | 2.152 ± 0.328 |
| Ketons | 0.785 ± 0.181 |  | 0.003 ± 0.002 |  | 0.524 ± 0.035 |  | 0.554 ± 0.103 |  | 0.599 ± 0.086 |
| Primary alcohols | 1.032 ± 0.254 |  | 0.709 ± 0.191 |  | 0.793 ± 0.138 |  | 0.889 ± 0.168 |  | 1.103 ± 0.180 |
| Aldehydes | 0.288 ± 0.089 |  | 0.321 ± 0.066 |  | 0.238 ± 0.057 |  | 0.233 ± 0.048 |  | 0.270 ± 0.046 |
| Fatty acids | 0.191 ± 0.066 |  | 0.166 ± 0.091 |  | 0.133 ± 0.024 |  | 0.122 ± 0.030 |  | 0.137 ± 0.045 |
| Isoalcohols | 0.393 ± 0.097 |  | 0.361 ± 0.111 |  | 0.281 ± 0.078 |  | 0.296 ± 0.040 |  | 0.346 ± 0.040 |
| Unknown | 0.784 ± 0.154 |  | 0.599 ± 0.058 |  | 0.576 ± 0.014 |  | 0.624 ± 0.117 |  | 0.886 ± 0.267 |

| **Supplementary Table 4**. Individual wax constituents of inflorescence stems of different Arabidopsis lines | | | | | | | | | | |
| --- | --- | --- | --- | --- | --- | --- | --- | --- | --- | --- |
| Wax contents | Composition | WT (μg/cm^2^) |  | cer1 (μg/cm^2^) |  | T-3 (μg/cm^2^) |  | T-8 (μg/cm^2^) |  | T-19 (μg/cm^2^) |
|  |  | Mean ± SD |  | Mean ± SD |  | Mean ± SD |  | Mean ± SD |  | Mean ± SD |
| n-Alkanes | C25 | 0.007 ± 0.003 |  | 0.004 ± 0.001 |  | 0.004 ± 0.000 |  | 0.005 ± 0.001 |  | 0.008 ± 0.004 |
|  | C26 | 0.010 ± 0.003 |  | 0.006 ± 0.001 |  | 0.006 ± 0.000 |  | 0.007 ± 0.001 |  | 0.011 ± 0.004 |
|  | C27 | 0.042 ± 0.010 |  | 0.006 ± 0.001 |  | 0.027 ± 0.000 |  | 0.028 ± 0.004 |  | 0.037 ± 0.001 |
|  | C28 | 0.015 ± 0.003 |  | 0.003 ± 0.001 |  | 0.010 ± 0.001 |  | 0.010 ± 0.002 |  | 0.013 ± 0.001 |
|  | C29 | 2.666 ± 0.467 |  | 0.023 ± 0.004 |  | 1.952 ± 0.099 |  | 2.053 ± 0.398 |  | 2.116 ± 0.296 |
|  | C30 | 0.037 ± 0.006 |  | 0.010 ± 0.012 |  | 0.026 ± 0.001 |  | 0.026 ± 0.005 |  | 0.027 ± 0.004 |
|  | C31 | 0.341 ± 0.074 |  | 0.092 ± 0.008 |  | 0.234 ± 0.014 |  | 0.233 ± 0.032 |  | 0.259 ± 0.037 |
|  | C32 | 0.127 ± 0.023 |  | 0.015 ± 0.001 |  | 0.097 ± 0.007 |  | 0.097 ± 0.023 |  | 0.095 ± 0.009 |
|  | C33 | 0.027 ± 0.012 |  | 0.019 ± 0.012 |  | 0.013 ± 0.002 |  | 0.014 ± 0.003 |  | 0.016 ± 0.005 |
|  | C34 | 0.024 ± 0.007 |  | 0.038 ± 0.017 |  | 0.027 ± 0.009 |  | 0.019 ± 0.007 |  | 0.024 ± 0.006 |
|  |  |  |  |  |  |  |  |  |  |  |
| Second-alcohols | C27 | 0.008 ± 0.002 |  | 0.002 ± 0.000 |  | 0.006 ± 0.000 |  | 0.006 ± 0.001 |  | 0.007 ± 0.001 |
|  | C29 | 2.701 ± 0.422 |  | 0.010 ± 0.004 |  | 2.090 ± 0.164 |  | 2.173 ± 0.471 |  | 2.145 ± 0.327 |
|  |  |  |  |  |  |  |  |  |  |  |
| Ketons | C29 | 0.785 ± 0.181 |  | 0.003 ± 0.002 |  | 0.524 ± 0.035 |  | 0.554 ± 0.103 |  | 0.599 ± 0.086 |
|  |  |  |  |  |  |  |  |  |  |  |
| Primary alcohols | C22 | 0.038 ± 0.010 |  | 0.023 ± 0.003 |  | 0.020 ± 0.009 |  | 0.023 ± 0.002 |  | 0.039 ± 0.017 |
|  | C24 | 0.017 ± 0.001 |  | 0.013 ± 0.004 |  | 0.013 ± 0.002 |  | 0.011 ± 0.001 |  | 0.018 ± 0.005 |
|  | C26 | 0.188 ± 0.042 |  | 0.133 ± 0.038 |  | 0.147 ± 0.026 |  | 0.169 ± 0.030 |  | 0.208 ± 0.031 |
|  | C28 | 0.486 ± 0.130 |  | 0.245 ± 0.066 |  | 0.369 ± 0.069 |  | 0.413 ± 0.075 |  | 0.510 ± 0.077 |
|  | C30 | 0.319 ± 0.080 |  | 0.295 ± 0.078 |  | 0.255 ± 0.042 |  | 0.283 ± 0.059 |  | 0.342 ± 0.065 |
|  | C32 | 0.017 ± 0.005 |  | 0.020 ± 0.022 |  | 0.007 ± 0.001 |  | 0.010 ± 0.003 |  | 0.016 ± 0.007 |
|  |  |  |  |  |  |  |  |  |  |  |
| Aldehydes | C24 | 0.006 ± 0.002 |  | 0.007 ± 0.001 |  | 0.003 ± 0.000 |  | 0.003 ± 0.001 |  | 0.004 ± 0.001 |
|  | C26 | 0.008 ± 0.001 |  | 0.005 ± 0.001 |  | 0.006 ± 0.000 |  | 0.006 ± 0.001 |  | 0.008 ± 0.002 |
|  | C28 | 0.032 ± 0.013 |  | 0.019 ± 0.006 |  | 0.024 ± 0.003 |  | 0.026 ± 0.005 |  | 0.032 ± 0.007 |
|  | C30 | 0.201 ± 0.065 |  | 0.255 ± 0.053 |  | 0.166 ± 0.047 |  | 0.163 ± 0.034 |  | 0.185 ± 0.041 |
|  | C32 | 0.020 ± 0.007 |  | 0.016 ± 0.005 |  | 0.015 ± 0.012 |  | 0.015 ± 0.008 |  | 0.021 ± 0.007 |
|  |  |  |  |  |  |  |  |  |  |  |
| Fatty acids | C22:0 | 0.007 ± 0.003 |  | 0.005 ± 0.001 |  | 0.005 ± 0.003 |  | 0.005 ± 0.002 |  | 0.007 ± 0.002 |
|  | C24:0 | 0.006 ± 0.003 |  | 0.005 ± 0.002 |  | 0.005 ± 0.001 |  | 0.004 ± 0.002 |  | 0.004 ± 0.002 |
|  | C26:0 | 0.073 ± 0.025 |  | 0.089 ± 0.053 |  | 0.037 ± 0.004 |  | 0.024 ± 0.006 |  | 0.025 ± 0.006 |
|  | C28:0 | 0.058 ± 0.012 |  | 0.016 ± 0.005 |  | 0.045 ± 0.008 |  | 0.052 ± 0.009 |  | 0.061 ± 0.011 |
|  | C30:0 | 0.046 ± 0.029 |  | 0.050 ± 0.030 |  | 0.041 ± 0.011 |  | 0.037 ± 0.013 |  | 0.040 ± 0.027 |
|  |  |  |  |  |  |  |  |  |  |  |
| Branch-alcohols | C28 | 0.069 ± 0.016 |  | 0.039 ± 0.006 |  | 0.051 ± 0.012 |  | 0.053 ± 0.004 |  | 0.065 ± 0.007 |
|  | C30 | 0.311 ± 0.082 |  | 0.309 ± 0.100 |  | 0.221 ± 0.064 |  | 0.236 ± 0.036 |  | 0.272 ± 0.035 |
|  | C32 | 0.013 ± 0.003 |  | 0.014 ± 0.006 |  | 0.009 ± 0.003 |  | 0.007 ± 0.001 |  | 0.009 ± 0.003 |

| **Supplementary Table 5**. Cuticular wax composition of rosette leaves of different Arabidopsis lines | | | | | | | | | |
| --- | --- | --- | --- | --- | --- | --- | --- | --- | --- |
| Wax | WT (μg/cm^2^) |  | cer1 (μg/cm^2^) |  | T-3 (μg/cm^2^) |  | T-8 (μg/cm^2^) |  | T-19 (μg/cm^2^) |
|  | Mean ± SD |  | Mean ± SD |  | Mean ± SD |  | Mean ± SD |  | Mean ± SD |
| Total load | 0.160 ± 0.025 |  | 0.095 ± 0.024 |  | 0.146 ± 0.017 |  | 0.189 ± 0.030 |  | 0.153 ± 0.010 |
| n-Alkanes | 0.077 ± 0.009 |  | 0.019 ± 0.004 |  | 0.070 ± 0.010 |  | 0.086 ± 0.012 |  | 0.068 ± 0.008 |
| Primary alcohols | 0.036 ± 0.013 |  | 0.028 ± 0.005 |  | 0.032 ± 0.005 |  | 0.044 ± 0.005 |  | 0.036 ± 0.005 |
| Aldehydes | 0.008 ± 0.000 |  | 0.006 ± 0.001 |  | 0.007 ± 0.001 |  | 0.008 ± 0.001 |  | 0.007 ± 0.000 |
| Fatty acids | 0.010 ± 0.002 |  | 0.010 ± 0.003 |  | 0.010 ± 0.001 |  | 0.014 ± 0.004 |  | 0.011 ± 0.003 |
| Isoalcohols | 0.005 ± 0.002 |  | 0.005 ± 0.002 |  | 0.004 ± 0.001 |  | 0.007 ± 0.002 |  | 0.005 ± 0.001 |
| Unknown | 0.025 ± 0.003 |  | 0.026 ± 0.009 |  | 0.022 ± 0.003 |  | 0.030 ± 0.006 |  | 0.025 ± 0.003 |

| **Supplementary Table 6**. Individual wax constituents of rosette leaves of different Arabidopsis lines | | | | | | | | | | |
| --- | --- | --- | --- | --- | --- | --- | --- | --- | --- | --- |
| Wax contents | Composition | WT (μg/cm^2^) |  | cer1 (μg/cm^2^) |  | T-3 (μg/cm^2^) |  | T-8 (μg/cm^2^) |  | T-19 (μg/cm^2^) |
|  |  | Mean ± SD |  | Mean ± SD |  | Mean ± SD |  | Mean ± SD |  | Mean ± SD |
| n-Alkanes | C25 | 0.001 ± 0.000 |  | 0.002 ± 0.000 |  | 0.001 ± 0.000 |  | 0.001 ± 0.000 |  | 0.002 ± 0.000 |
|  | C26 | 0.002 ± 0.000 |  | 0.002 ± 0.001 |  | 0.002 ± 0.000 |  | 0.002 ± 0.000 |  | 0.002 ± 0.000 |
|  | C27 | 0.002 ± 0.001 |  | 0.001 ± 0.001 |  | 0.002 ± 0.000 |  | 0.003 ± 0.001 |  | 0.002 ± 0.000 |
|  | C28 | 0.001 ± 0.000 |  | 0.001 ± 0.000 |  | 0.001 ± 0.000 |  | 0.001 ± 0.000 |  | 0.001 ± 0.000 |
|  | C29 | 0.019 ± 0.003 |  | 0.004 ± 0.001 |  | 0.016 ± 0.002 |  | 0.020 ± 0.005 |  | 0.014 ± 0.002 |
|  | C30 | 0.002 ± 0.000 |  | 0.001 ± 0.000 |  | 0.001 ± 0.000 |  | 0.002 ± 0.001 |  | 0.002 ± 0.000 |
|  | C31 | 0.033 ± 0.004 |  | 0.005 ± 0.001 |  | 0.032 ± 0.006 |  | 0.038 ± 0.006 |  | 0.030 ± 0.005 |
|  | C32 | 0.001 ± 0.000 |  | 0.000 ± 0.000 |  | 0.001 ± 0.001 |  | 0.002 ± 0.000 |  | 0.002 ± 0.000 |
|  | C33 | 0.013 ± 0.002 |  | 0.002 ± 0.001 |  | 0.012 ± 0.002 |  | 0.014 ± 0.001 |  | 0.013 ± 0.002 |
|  | C34 | 0.000 ± 0.000 |  | 0.000 ± 0.000 |  | 0.000 ± 0.000 |  | 0.001 ± 0.000 |  | 0.000 ± 0.000 |
|  | C35 | 0.002 ± 0.001 |  | 0.001 ± 0.000 |  | 0.001 ± 0.000 |  | 0.001 ± 0.001 |  | 0.001 ± 0.001 |
|  |  |  |  |  |  |  |  |  |  |  |
| Primary alcohols | C22 | 0.001 ± 0.000 |  | 0.001 ± 0.001 |  | 0.001 ± 0.001 |  | 0.002 ± 0.000 |  | 0.001 ± 0.001 |
|  | C24 | 0.001 ± 0.000 |  | 0.001 ± 0.000 |  | 0.001 ± 0.000 |  | 0.001 ± 0.000 |  | 0.001 ± 0.000 |
|  | C26 | 0.002 ± 0.000 |  | 0.003 ± 0.001 |  | 0.002 ± 0.000 |  | 0.003 ± 0.001 |  | 0.002 ± 0.000 |
|  | C28 | 0.009 ± 0.001 |  | 0.008 ± 0.002 |  | 0.009 ± 0.003 |  | 0.011 ± 0.003 |  | 0.010 ± 0.001 |
|  | C30 | 0.007 ± 0.001 |  | 0.007 ± 0.001 |  | 0.009 ± 0.003 |  | 0.014 ± 0.003 |  | 0.010 ± 0.002 |
|  | C32 | 0.010 ± 0.001 |  | 0.008 ± 0.001 |  | 0.010 ± 0.001 |  | 0.012 ± 0.001 |  | 0.012 ± 0.003 |
|  | C34 | 0.001 ± 0.000 |  | 0.001 ± 0.000 |  | 0.001 ± 0.001 |  | 0.002 ± 0.001 |  | 0.001 ± 0.000 |
|  |  |  |  |  |  |  |  |  |  |  |
| Aldehydes | C24 | 0.001 ± 0.000 |  | 0.001 ± 0.000 |  | 0.001 ± 0.000 |  | 0.001 ± 0.000 |  | 0.001 ± 0.000 |
|  | C26 | 0.001 ± 0.000 |  | 0.001 ± 0.000 |  | 0.001 ± 0.000 |  | 0.001 ± 0.000 |  | 0.001 ± 0.000 |
|  | C28 | 0.002 ± 0.000 |  | 0.001 ± 0.000 |  | 0.002 ± 0.001 |  | 0.002 ± 0.001 |  | 0.002 ± 0.001 |
|  | C30 | 0.001 ± 0.000 |  | 0.000 ± 0.000 |  | 0.001 ± 0.000 |  | 0.001 ± 0.000 |  | 0.001 ± 0.000 |
|  | C32 | 0.004 ± 0.000 |  | 0.003 ± 0.001 |  | 0.003 ± 0.000 |  | 0.003 ± 0.000 |  | 0.003 ± 0.001 |
|  |  |  |  |  |  |  |  |  |  |  |
| Fatty acids | C22:0 | 0.001 ± 0.000 |  | 0.001 ± 0.000 |  | 0.001 ± 0.000 |  | 0.001 ± 0.000 |  | 0.001 ± 0.000 |
|  | C24:0 | 0.000 ± 0.000 |  | 0.000 ± 0.000 |  | 0.001 ± 0.000 |  | 0.001 ± 0.000 |  | 0.000 ± 0.000 |
|  | C26:0 | 0.002 ± 0.001 |  | 0.002 ± 0.001 |  | 0.002 ± 0.000 |  | 0.002 ± 0.000 |  | 0.002 ± 0.001 |
|  | C28:0 | 0.002 ± 0.001 |  | 0.002 ± 0.000 |  | 0.002 ± 0.000 |  | 0.004 ± 0.001 |  | 0.003 ± 0.000 |
|  | C30:0 | 0.004 ± 0.001 |  | 0.004 ± 0.002 |  | 0.005 ± 0.001 |  | 0.006 ± 0.002 |  | 0.005 ± 0.002 |
|  |  |  |  |  |  |  |  |  |  |  |
| Branch-alcohols | C28 | 0.001 ± 0.000 |  | 0.001 ± 0.000 |  | 0.002 ± 0.000 |  | 0.002 ± 0.001 |  | 0.001 ± 0.000 |
|  | C30 | 0.003 ± 0.001 |  | 0.004 ± 0.002 |  | 0.003 ± 0.001 |  | 0.004 ± 0.002 |  | 0.003 ± 0.001 |
|  | C32 | 0.001 ± 0.001 |  | 0.000 ± 0.000 |  | 0.000 ± 0.000 |  | 0.001 ± 0.000 |  | 0.001 ± 0.000 |

| **Supplementary Table 7**. Sequences of primers used in cloning and PCR reactions | | |
| --- | --- | --- |
| Gene | Forward (5’—3’) | Reverse (5’—3’) |
|  | Primers used for qRT-PCR analysis | |
| *BdCER1-1* | AAGGGGCTCCTCGTCGG | GAGCGCGTGGTAGCAGGA |
| *BdCER1-2* | GGCCACACATATAACATGCAGGT | GCATCTGACAAGAATGGATCGAGTA |
| *BdCER1-3* | GTCGACCGGATCGACCAG | ACCGAACAAACGAGCCGC |
| *BdCER1-4* | GACATGGTCGGCTGCTACAA | TACTGTACTACTTCGGTTCAATTGC |
| *BdCER1-5* | CCCGGGTTCGCTGAGTAT | TGGATCCTTAGTTTCTTCATCACA |
| *BdCER1-6* | CAAACAAGGTCGTCTCCGTGC | GGCTGCAGGACCAATCGC |
| *BdCER1-7* | CTTAAGAAGACACGCAAGGATGAT | CCAACCTTCCAGAGCATGTACC |
| *BdCER1-8* | TGCGTTGGAGGGATGGAAG | GAGCATCAAGCATGAGCAACG |
| *BdActin* | GACCTTCAACACTCCTGCTATG | CGCCAGAATCCAGCACAATA |
| *AtCER1* | AAGGATGGGAAATGCATGAG | TGATGTGGAAGGAGGAGAGG |
| *AtACT8* | CCGAGCAGCATGAAGATTAAG | CATACTCTGCCTTAGAGATCCACA |
|  | Primers used for Semi-qPCR analysis | |
| *BdCER1-8* | TTAGCCGCTGCTGTGGTTGT | TCCGCACTCGTCTTCCTTCC |
| *AtACT8* | AAAGATGCGTATGTTGGTGA | ACAATTTCCCGTTCTGCTGT |
|  | Primers used for cloning of full length CDS | |
| *BdCER1-8* | GAGTAGTTTGCAATGGCT | TGACTTACTGACCACAACC |
|  | Primers used for checking hygromycin gene | |
| *Hyg* | ATGTTGGCGACCTCGTATT | CGTTATGTTTATCGGCACTTT |

Supplementary Data Set 1

| gene name | protein sequence |
| --- | --- |
| *AtCER1* | MATKPGVLTDWPWTPLGSFKYIVIAPWAVHSTYRFVTDDPEKRDLGYFLVFPFLLFRILHNQVWISLSRYYTSSGKRRIVDKGIDFNQVDRETNWDDQILFNGVLFYIGINLLPEAKQLPWWRTDGVLMAALIHTGPVEFLYYWLHKALHHHFLYSRYHSHHHSSIVTEPITSVIHPFAEHIAYFILFAIPLLTTLLTKTASIISFAGYIIYIDFMNNMGHCNFELIPKRLFHLFPPLKFLCYTPSYHSLHHTQFRTNYSLFMPLYDYIYGTMDESTDTLYEKTLERGDDIVDVVHLTHLTTPESIYHLRIGLASFASYPFAYRWFMRLLWPFTSLSMIFTLFYARLFVAERNSFNKLNLQSWVIPRYNLQYLLKWRKEAINNMIEKAILEADKKGVKVLSLGLMNQGEELNRNGEVYIHNHPDMKVRLVDGSRLAAAVVINSVPKATTSVVMTGNLTKVAYTIASALCQRGVQVSTLRLDEYEKIRSCVPQECRDHLVYLTSEALSSNKGFWVKVWLVGEGTTREEQEKATKGTLFIPFSQFPLKQLRRDCIYHTTPALIVPKSLVNVHSCENWLPRKAMSATRVAGILHALEGWEMHECGTSLLLSDLDQVWEACLSHGFQPLLLPHH |
| *AtCER3* | MVAFLSAWPWENFGNLKYLLYAPLAAQVVYSWVYEEDISKVLWCIHILIICGLKALVHELWSVFNNMLFVTRTLRINPKGIDFKQIDHEWHWDNYIILQAIIVSLICYMSPPLMMMINSLPLWNTKGLIALIVLHVTFSEPLYYFLHRSFHRNNYFFTHYHSFHHSSPVPHPMTAGNATLLENIILCVVAGVPLIGCCLFGVGSLSAIYGYAVMFDFMRCLGHCNVEIFSHKLFEILPVLRYLIYTPTYHSLHHQEMGTNFCLFMPLFDVLGDTQNPNSWELQKKIRLSAGERKRVPEFVFLAHGVDVMSAMHAPFVFRSFASMPYTTRIFLLPMWPFTFCVMLGMWAWSKTFLFSFYTLRNNLCQTWGVPRFGFQYFLPFATKGINDQIEAAILRADKIGVKVISLAALNKNEALNGGGTLFVNKHPDLRVRVVHGNTLTAAVILYEIPKDVNEVFLTGATSKLGRAIALYLCRRGVRVLMLTLSMERFQKIQKEAPVEFQNNLVQVTKYNAAQHCKTWIVGKWLTPREQSWAPAGTHFHQFVVPPILKFRRNCTYGDLAAMKLPKDVEGLGTCEYTMERGVVHACHAGGVVHMLEGWKHHEVGAIDVDRIDLVWEAAMKYGLSAVSSLTN |
| *CsCER1* | MASKPGILTDWPWKPLGSFKFVILTPWVIHSSYLYFKGGEKRDLSYILIFPFLVLRMIHNQIWISLSRYQTAKGTKRIVDKPIEFEQVDRESSWDDQILFNGLLFCLGRMVVEKGENLPLWRTNGVVIAALLHAGPVEFLYYWFHRALHHHFLYSRYHSHHHSSIATEPITSVIHPFAEHIVYFLLFTIPLLVTVLTETASIGSFVLYVMFIDFMNNMGHCNFEIVPKSLFFIFPPLKYLIYTPSFHSLHHTQFRTNYSLFMPIYDYIYGTVDKNSDSLYENSLLREEEVADVVHLSHLTTPQSIYHMRLGLATVASQPFTSKWWLTLLWPFTSFYVLATSFYGHIFVYERNTFKALKLQSWVIPRFNLQYFMKGRREAINKLIEAAILDADKKGVKGKELNEYGEFYIHKYPNLRIKLVDGSSLAAAIVINTIPKATTKVLLRGNLSKVAYAIADALCQLGFQVATLYENEHKKLKSKVTTNSNNLVLAKITTHKIWIVGDGLEEFEQLNAPKGTIFIPYSQFPPQRLRKDCYYHITPSMRVPSSFQNIDSCENWLPRRVMSAWRMAGILHALEGREGHECGETMLSLDDAWRASLENGFLPLEIPSI* |
| *CsCER3* | MVAPLASWPWENLGMFKYLLYGPLLANGLYTLYEEGNIIHNWCLHILLISLLRVGIHVVWSSYSNMLFLTRNRRILQQGVDFKQIDMEWEWDNFLLLQALMTSMMVYLFPSLGNLPLWNPKGLIAVLILHIVIAEPLFYFFHRLFHSNHYLFTHYHSLHHSSSVPQSFTAGNGTVLEHLAWSIVIGAPIVGTSLLGYGSTATFACYVLVFDFLRCLGLSNVEIVSHRLFDAIPVLRYLLYTPTYHTLHHTEKETNFCLFMPLFDAIGNTLHKCSWKLHKQNSLNAGKNGRVPDFVFLAHVVDVTSSMHAPFVSRFFASRPFVTKLSLFPSWPAAFIVMLIMWGRSKIFLYSYYNLRNWLHQTWVVPRFGFQYFLPFAREGINKHIEDAILRADKLGVKVISLAALNKNEALNGGGTLFVEKHPNLRVRVVHGNTLTAAVILNEIPKDVKEVFLTGATSKLGRAIALYLCRRKVRVLMLTLSTERFEKIQKEAPVDCQNYLVQVTKYQAARNCKTWIVGKWITPREQSWAPSGTHFHQFVVPPILAFRRDCTYGDLAAMRLPEDVQGLGNCEYTMSRGVVHACHAGGVVHHLEGWTHHEVGALDVDRIDLVWEAALKHGLKPVSTK |
| *BnCER1* | MATKPGILTDWPWTPLGNFKYIVIAPWAVHSTYKFVTDDPVDLGYSLVLPFLLFRILHNQVWISLSRYYTTKGKRRILDKGIDFNQVDRETNWDDQILFNGLLFYIGIMLLPQAKQLPWWRTDGVLMAAMLHAGPVEFLYYWLHKALHHHFLYSRYHSHHHSSIVTEPITSVIHPFAEHIAYFILFAIPLLTTLLTKTASIASFSGYVIYIDFMNNMGHCNFELVPKRLFHLFPPLKYLCYTPSFHSLHHTQFRTNYSLFMPLYDYIYGTMDETSDTLYEKSLERGEDRVDVVHLTHLTTPESIYHLRIGLASFASYPFSYRWFMRLLWPFTSLSMLFTLFYASLFVSERNSFEKLNLQSWIIPRYNLQYLLKWRKDAINNMIEKAILEANEKGVKVLSLGLMNQGEELNRNGEVYIHKHPEMKVRVVDGSRLTAAVVINSLPKSTTKIVMTGNLTKVAYTIASALCQRGVEVLTLLPEEYEKLSSFVPKECRDRLILLTSETLASNKVWLMGEGTTREEQEMATKGTLFIPFSQFPLKQLRRDCIYHTPPALIIPKSLVNIHSCENWLPRKAMSATRVAGILHALEGWETHECGTSNILLSDLDQVWEACLSHGFQPLLLPHHFQYP |
| *OsWDA1* | MATNPGLFTEWPWKKLGSFKYVLLAPWVAHGWYEVATKGWREVDLGYIAILPSLLLRMLHNQAWITISRLQNARGRRQIVRRGIEFDQVDRERNWDDQIILSGILLYLGALYVPGGQHLPLWRTDGAGLIALLHAGPVEFLYYWFHRALHHHFLYTHYHSHHHSSIVTEPITSVIHPFAELVAYELLFSIPLIACALTGTASIIAFEMYLIYIDFMNNMGHCNFELVPSWLFTWFPPLKYLMYTPSFHSLHHTQFRTNYSLFMPFYDYIYNTMDKSSDTLYENSLKNNEEEEAVDVVHLTHLTTLHSIYHMRPGFAEFASRPYVSRWYMRMMWPLSWLSMVLTWTYGSSFTVERNVMKKIRMQSWAIPRYSFHYGLDWEKEAINDLIEKAVCEADKNGAKVVSLGLLNQAHTLNKSGEQYLLKYPKLGARIVDGTSLAAAVVVNSIPQGTDQVILAGNVSKVARAVAQALCKKNIKVTMTNKQDYHLLKPEIPETVADNLSFSKTGTAKVWLIGDGLDSAEQFRAQKGTLFIPYSQFPPKMVRKDSCSYSTTPAMAVPKTLQNVHSCENWLPRRVMSAWRIAGILHALEGWNEHECGDKVLDMDKVWSAAIMHGFCPVAQG |
| *OsCER1* | MATRPGPLTEWPWHRLGNFKYVVMAPVVAHGARRVMRNGWGDLDIAFSLILPSLLLRMIHNQIWISLSRYQTARSKHRIVDRGIEFDQVDRERGWDDQILFNGLVFYAGYLAMPSVRRMPVWRTDGAVVTALVHTGPVEFLYYWFHRALHHHFLYSRYHSHHHASIVTEPITSVIHPFAEHVVYFILFAIPILSTIYLGNVSAMGIVGYIAYIDFMNNMGHCNFELVPEWIFQIFPPLKYLIYTPSFHSLHHTQFRTNYSLFMPFYDYIYNTMDKSSDELYESSLKGTEETPDLVHLTHMTNLQSAYHLRIGIASIASKPYSDSAWYMWTLWPLAWLSMVLAWIYGSSAFVVERIKLNKMKMQTWALPRYNFQYGLTWEREPINDLIEKAILDADMKGVKVISLGLLNQAKQLNGNGELFRQKYPKLGVRIIDGSGLATAVVLKSIPSDAKKVFLRTGTSKIARAIAIALCDRGVQVIMNEKEVYHMLKSQIPENRASYLKLSSDNVPQLWIVHNIDDNEQKMAPKGTIFIPISQFPLKKLRKDCTYMSTPAMRIPEEMKNIHSCENWLPRRVMSAWHIAGILHALEGWNMHECGDEMMDIEKSWSAAIRHGFLPLTKA |
| *ZmGL1* | MGAALLASWPWDNLGFYKYVLYGPLVGKAVASRAWEAASPDRWILLLLLLFGLRALTYQLWSSFSNMLFATRRRRVVRDGVDFDQIDKEWDWDNFLILHALMAAAALCAFPSLRHLPAWDGRGFAVALVAHAAATEPLSYLAHRALHGSSGRLYARYHSLHHSSRVPQPFTAGLATPLEHVALGALMSLPLAAARAAGCASVALAFAYVLAFDSLRAMGHCNVEVVPASLFRAIPALRYVLYTPTYHAIHHTKKEANFCLFMPLFDLLGGTIDRRSWDMQRKMSAGVDEVPDFVFLAHVVDVMQSLHVPFVMRTFASTPFSVQLFLLPMWPFAFLVMLAMWVWSKTFVISCYNLRGRLHQIWAVPRYGFQYFLPFAKDGINRQIELAILRADKMGVKVLSLAALNKNEALNGGGTLFVNKHPDLRVRVVHGNTLTAAVILNEIPKGTAEVFLTGATSKLGRAIALYLCKKRVRVMMMTLSTERFQKIQKEAPAEFQQYLVQVTKYRSAQHCRTWIVGKWLSPREQRWAPPGTHFHQFVVPPIIGFRRDCTYGKLAAMRLPKDVRGLGACEYSLERGVVHACHAGGVVHFLEGYTHHEVGAIDVDRIDVVWEAALKHGLRPA |
| *OsGL1-1* | MGAAFLSSWPWDNLGAYKYVLYAPLVGKAVAGRAWERASPDHWLLLLLVLFGVRALTYQLWSSFSNMLFATRRRRIVRDGVDFGQIDREWDWDNFLILQVHMAAAAFYAFPSLRHLPLWDARGLAVAALLHVAATEPLFYAAHRAFHRGHLFSCYHLQHHSAKVPQPFTAGFATPLEQLVLGALMAVPLAAACAAGHGSVALAFAYVLGFDNLRAMGHCNVEVFPGGLFQSLPVLKYLIYTPTYHTIHHTKEDANFCLFMPLFDLIGGTLDAQSWEMQKKTSAGVDEVPEFVFLAHVVDVMQSLHVPFVLRTFASTPFSVQPFLLPMWPFAFLVMLMMWAWSKTFVISCYRLRGRLHQMWAVPRYGFHYFLPFAKDGINNQIELAILRADKMGAKVVSLAALNKNEALNGGGTLFVNKHPGLRVRVVHGNTLTAAVILNEIPQGTTEVFMTGATSKLGRAIALYLCRKKVRVMMMTLSTERFQKIQREATPEHQQYLVQVTKYRSAQHCKTWIVGKWLSPREQRWAPPGTHFHQFVVPPIIGFRRDCTYGKLAAMRLPKDVQGLGACEYSLERGVVHACHAGGVVHFLEGYTHHEVGAIDVDRIDVVWEAALRHGLRPV |
| *OsGL1-2* | MAAPPLSSWPWASLGSYKYVLYGAVVWKVAEEWRQQGAAPVGSWWLHLLLLFAARGLTYQFWFSYGNMLFFTRRRRVVPDSVDFRQVDAEWDWDNFLLLQTLIGATLVGSPAVARQQLLLPSLKQAWDPRGWAIALLLHVLVAEPLFYWAHRALHRAPLFSRYHAAHHHASVTTPLTAGFGTPLESLLLTVVIGVPLAGAFLMGVGSVGLVYGHVLLFDFLRSMGYSNVEVISPRVFQAVPLLRYLIYTPTYLSLHHREKDSNFCLFMPIFDLLGGTLNHKSWELQKEVYLGKNDQAPDFVFLAHVVDIMASMHVPFVLRSCSSTPFANHFVLLPFWPVAFGFMLLMWCCSKTFLVSSYRLRGNLHQMWTVPRYGFQYFIPAAKKGINEQIELAILRADRMGVKVLSLAALNKNEALNGGGTLFVNKHPELRVRVVHGNTLTAAVILNEIPSNVKDVFLTGATSKLGRAIALYLCRKKIRVLMLTLSSERFLKIQREAPAEFQQYLVQVTKYQPAQNCKTWLVGKWLSPREQRWAPAGTHFHQFVVPPIIGFRRDCTYGKLAAMRLPKDVQGLGYCEYTMERGVVHACHAGGVVHFLEGWEHHEVGAIDVDRIDVVWKAALKHGLTPA |
| *OsGL1-3* | MAISMASPLSSWPWAFLGSYKYLLYGPVVGKVVQEWREQGRLPLGTSWCLHLILLLALRSLTYQLWFSYGNMLFFTRRRRVVDDGVDFRQIDTEWDWDNMVIMQTLIAAVLVTSRVFPATSDLSAWDLRGWAIAVVLHVAVSEPAFYWAHRALHLGPLFSRYHSLHHSFQATQALTAGFVTPLESLILTLVAWAPLAGAFMAGHGSVSLVYGHILLFDYLRSMGYSNVEVISHKTFQDFPFLRYLIYTPSYLSLHHREKDSNFCLFMPLFDALGGTLNPKSWQLQKEVDLGKNHRVPDFVFLVHVVDVVSSMHVPFAFRACSSLPFATHLVLLPLWPIAFGFMLLQWFCSKTFTVSFYKLRGFLHQTWSVPRYGFQYFIPSAKKGINEMIELAILRADKMGVKVLSLAALNKNEALNGGGTLFVRKHPDLRVRVVHGNTLTAAVILNEIPGDVAEVFLTGATSKLGRAIALYLCRKKIRVLMLTLSTERFMNIQREAPAEFQQYLVQVTKYQAAQNCKTWIVGKWLSPREQRWAPAGTHFHQFVVPPIIGFRRDCTYGKLAAMRLPEDVEGLGTCEYTMGRGVVHACHAGGVVHFLEGWDHHEVGAIDVDRIDAVWNAALRHGLTPA |
| *OsGL1-4* | MATRPGPLTEWPWHRLGNFKYVVMAPVVAHGARRVMRNGWGDLDIAFSLILPSLLLRMIHNQIWISLSRYQTARSKHRIVDRGIEFDQVDRERGWDDQILFNGLVFYAGYLAMPSVRRMPVWRTDGAVVTALVHTGPVEFLYYWFHRALHHHFLYSRYHSHHHASIVTEPITSVIHPFAEHVVYFILFAIPILSTIYLGNVSAMGIVGYIAYIDFMNNMGHCNFELVPEWIFQIFPPLKYLIYTPSFHSLHHTQFRTNYSLFMPFYDYIYNTMDKSSDELYESSLKGTEETPDLVHLTHMTNLQSAYHLRIGIASIASKPYSDSAWYMWTLWPLAWLSMVLAWIYGSSAFVVERIKLNKMKMQTWALPRYNFQYGLTWEREPINDLIEKAILDADMKGVKVISLGLLNQAKQLNGNGELFRQKYPKLGVRIIDGSGLATAVVLKSIPSDAKKVFLRTGTSKIARAIAIALCDRGVQVIMNEKEVYHMLKSQIPENRASYLKLSSDNVPQLWIVHNIDDNEQKMAPKGTIFIPISQFPLKKLRKDCTYMSTPAMRIPEEMKNIHSCENWLPRRVMSAWHIAGILHALEGWNMHECGDEMMDIEKSWSAAIRHGFLPLTKA |
| *OsGL1-5* | MATNPGLFTEWPWKKLGSFKYVLLAPWVAHGWYEVATKGWREVDLGYIAILPSLLLRMLHNQAWITISRLQNARGRRQIVRRGIEFDQVDRERNWDDQIILSGILLYLGALYVPGGQHLPLWRTDGAGLIALLHAGPVEFLYYWFHRALHHHFLYTHYHSHHHSSIVTEPITSVIHPFAELVAYELLFSIPLIACALTGTASIIAFEMYLIYIDFMNNMGHCNFELVPSWLFTWFPPLKYLMYTPSFHSLHHTQFRTNYSLFMPFYDYIYNTMDKSSDTLYENSLKNNEEEEAVDVVHLTHLTTLHSIYHMRPGFAEFASRPYVSRWYMRMMWPLSWLSMVLTWTYGSSFTVERNVMKKIRMQSWAIPRYSFHYGLDWEKEAINDLIEKAVCEADKNGAKVVSLGLLNQAHTLNKSGEQYLLKYPKLGARIVDGTSLAAAVVVNSIPQGTDQVILAGNVSKVARAVAQALCKKNIKVTMTNKQDYHLLKPEIPETVADNLSFSKTGTAKVWLIGDGLDSAEQFRAQKGTLFIPYSQFPPKMVRKDSCSYSTTPAMAVPKTLQNVHSCENWLPRRVMSAWRIAGILHALEGWNEHECGDKVLDMDKVWSAAIMHGFCPVAQG |
| *OsGL1-6* | MASKPGPLTQWPWHNLGNYKYALVAPSAAYSTYRFVTASSAAERDLLNFMVFPMLLLRLLYGQLWITVSRHQTARSKHKIVNKSLDFEQIDRERNWDDQIILTALVFYLVSATMPQAQVAPWWSTKGMVVTAVLHAGPVEFLYYWLHRALHHHWLYARYHSHHHASIVTEPITSVIHPFAEEVVYFVLLAIPILSTVATGTVSVVTANGYLVYIDFMNYLGHCNFELVPKCLFHVFPPLKYLLYTPSFHSLHHTQFRTNYSLFMPVYDYIYGTTDKSSDELYERTLQGRDEAAWRPDVVHLTHLTTPESVFHNRLGFAAVASNPLGAAASGHLLRAASAVASPLLSLFASTFRSEANRLDKLNIETWVIPRFTSHYTSKSDGYKVSRLIEKAVSDAEASGARVLTLGLLNQGYDLNRNGELYVVRKPSLKTKIVDGTSLAVAAVLNMIPQGTKDVLLLGNANKISLVLTLSLCKREIQVRMVNKELYECLKQQLQPEMQEHLVLSCSYSSKVWLVGDGVTDEEQMKAQKGSHFVPYSQFPPNKARNDCVYHCTPALLVPESFENLHVCENWLPRRVMSAWRAAGIVHALEKWDGHECGGRVTGVQKAWSAALARGFRPYDDHHHPGITHDGRGGL |
| *OsGL1-7* | MGTGRDDQIILNGLLFYLGYAIIPNFRLMPVWRTNGALITILLHMGPVEFLYYWFHRALHHHFLYSRYHSHHHASIVTEPITSVIHPFAEHLAYFLLFSISILPPIFMGCGSVLAGVLYITYIDFMNNMGHCNFELMPKWMFQTFPPLKYLIYTPSFHSLHHTQFRTNYSLFMPFYDYIYNTMDSSSDELYERSLKGTEETPDIVHLTHMTSLKSTYHLRIGITSISSKPCNDSVWYMWMLWPVAWLSMVLAWIYGSSAFVVERLKLKKFSMQVWALPRYNFQVMDSSAAEQLNGSGELFAKKYPRLRVRLIDGSGLATAVVLNSIPFGTKQVFLCGSNSKVTRATAIALCQRGVQVILNQEKEYGMLKSRVPESRAIYLKFSNDETPQIWIGDSIDDAQGRAPKGTIFIPTSQFPLKKARKDCTYLSNPAMKIPETMQNVHTCENWLPRRVMSAWRIAGILHALEGWEMHECGDDMMTIEKTWSAAIKHGFKPLTKPCSLNSGTDL* |
| *BdCER1-1* | MGAAFLSSWPWDNLGLFKYALYGPLVGKAVASRAWERGSPDQWLLLLLVLFTLRAFTYQLWSSYSNMLFLTRRRRIVRDGVDFAQIDKEWDWDNFLILQILMAATAFYAFPSLRDLPLWDAKGLLVGALLHVVATEPLFYVAHRAFHSGHLFSCYHALHHSIKVPTPFTAGFATPLEHMVLGALMALPLAGACAAGHGSVGLAFAYVLGFDFLRAMGHCNVELFPAGIFQALPLLRYLIYTPTYHTVHHTEKDANFCLFMPLFDRLGGTLDANTWELQRKTRAGVDEVPDFVFLAHVVDVMQSMHVPFVMRTFSSTPFAVRAFLVPLWPIAFVFMLMVWAWSKTFVISYYHLRGKLHQMWAVPRYGFHYFLPFAKDGINYQIELAILRAERMGVKVVSLAALNKNEALNGGGTLFVNKHPDLRVRVVHGNTLTAAVILNEIPKGTTEVFMTGATSKLGRAIALYLCRKKIRVMMMTLSTERFQKIQKEAAAEHQQYLVQVTKYRSAGQCKTWIVGKWLSPREQRWAPPGTHFHQFVVPPILGFRRDCTYGKLAAMRLPKDARGLGSCEFSLERGVVHACHAGGVVHFLEGYTHHEVGAIDVGRIDVVWEAALKHGLRPA |
| *BdCER1-2* | MAPALSSWPWASLGVYKYFLLAPLAWKAAQEWRGKQGGGAVLPVDSWWLHLLALFWARGLTYQFWYSYSSMLFLTRRRRVVPDGVDFRQVDLEWDWDNFLLLQTLIGAVVVNGPLLPGPKHLSLWDPRGLAIVLLLHVGFSEPVFYLAHRALHGASLFRQYHAAHHSSRVTQPLTAGFGTPLEALLLMLTTGVPLAGAFLMGSGSIGLVYVYLLAFDYLRSMGYSNVEVISHRVFEALPPLRYLIYTPSYLSLHHREKDSNFCLFMPLYDLLGGTLNSKSWELQKETYLGKEERAPDFVFLVHVVDIMSSMHVPFVLRSISSVPFANHLVLLPFWPVAVAYMLLMWCCSKTFLVSFYYLRGRLHQTWSVPRHGFQYFIPAAKDGINRQIELAVLRADRIGVKVLSLAALNKNEALNGGGTLFVDKHPELRVRVVHGNTLTAAVILNEIPSNAKDVFLTGATSKLGRAIALYLCRKKIRVMMLTMSSERFLKIQREAPAEFQQYLVQVTKYQAAQNCKTWIVGKWLSPREQRWAPAGTHFHQFVVPPIIGFRRDCTYGKLAAMRLPKDVQGLGSCEYTMERGVVHACHAGGVVHFLEGWEHHEVGALDVDRIDVVWKAALKHGLTPA |
| *BdCER1-3* | MVSPLSSWPWAALGSYKYLLYGPVVAKAVQAWRDEESSWSSLMESWCVHLVLLLALRSLTYQLWFSYGNMLFLTRRRRVVPDGVDFQQIDAEWHWDNMVMMQTLIGAMVMNSSPFSSAGGLIRAWDPRGWAVALLLHVALSEPFFRWAHMALHRDPLFSRYHSKHHSSPVTQPLTAAYGTPLESLLLTLAMGVPLAGAFLAGSGSLSLVYGYVFLFDYLRCMGYSNVEVISHRAFQAFPPLRYLIYTPTYLSLHHKEKDCNYCLFMPLYDLLGGTLHRSSFTLQKEIDSGKNDRVPDFVFLAHVVDVVSSMHVPFAFRSCSSLPWSPHLVLLPLWPIALGIMLLQVLCSKTFTVSFYFLRGRLHQTWTIPRYSFQYFIPPMKKGINRQIELAILRADKMGVKVLSLAALNKNEALNGGGTLFVAKHPDLKVRVVHGNTLTAAVILNEIPSNVKEVFLTGATSKLGRAIALYLCRKKIRVLMLTLSSERFLKIQREAPAEFQPFLVQVTKYQAAKNCKTWLVGKWLSPREQRWAAAGTHFHQFVVPPVMEFRRDCSYGKLAAMRLPKDVQGLGSCEYTMERGVVHACHAGGVVHCLEGWEHHEVGAIDVDRIDQVWHAALKHGLSPP |
| *BdCER1-4* | MATRPGPLTEWPWQRLGNFKYLVMAPVVVHGAHRVMTKGWGDIDLAYALILPSLLLRMIHNQIWISLSRYQTARSKHRIVDRGIEFDQVDRERGWDDQIIFNGLLFYAGYLAMPSVRGFPLWRTDGAVMTALLHAGPVEFLYYWFHRALHHHFLYSRYHSHHHASIVTEPITSVIHPFGEHVVYFTLFAIPMLSTIYMGNGSALVFVLYIVYIDFMNNMGHCNFELVPKWAFQVFPPLKYLMYTPSFHSLHHTQFRTNYSLFMPFYDYIYSTMDKSSDELYENSLKGTEETPDLVHLTHMTNLQSAYHLRIGFASIASKPSENSEWYMWTLWPLAWLSMVVAWMYGSSAFVVERIKLKKLKMQTWVIPRYNFQYALTWERESINDLIEKAILDADVRGVKVLSLGLLNQTKELNGAGELFRQKYPKLGVQLVDGSGLATAVVLKSIPLDAKQVFLQTGTSKIARAIAITLCGRGIQVIMNRKKEYDILKPQIPENRASYLKCSSDDMPKIWLVDCIDAKEQLVAPKGTVFIPISQFPTKKVRKDCTYLSTPAMKIPEAMQNIHSCENWLPRRVMSAWHIAGILHVLEGWSMHECGDSMMDIEKTWSAATRHGFVPLTKA |
| *BdCER1-5* | MEQKPVFFLISVNTVSARSSPKGQHKADMATNPGFLSEWPWKRLGSFKYLVLAPWVAHGFHQLATKGWRETDLGYIAILPSMLLRALHNQAWITVSRLQNARGRKQIVDRGIEFEQVDRERNWDDQIILSAILFYLGALHLPGGQHLPLWRTDGAVLVALLHAGPVELLYYWFHRALHHHFLYTRYHSHHHASIVTEPITSVIHPFAELVAYELLFSIPMIASALTGTASIVAFEMYMLYIDFMNNMGHCNFELVPTWLFRWFPPLKYFMYTPSFHSLHHTQFRTNYSLFMPFYDYIYNTMDKSSDKLHENSLKNKEEAVDVVHLTHLTSLQSIYHMRPGFAEYASKPYTSKWYMRIMWPLSWLSMVLTWVYGSWFTVERNVMKKLRIQSWAIPRYNFHYGLNWEKEAINSLIIKAIHEADKNGAKVVSLGLLNQAQSLNGSGELYLQKYPKLGVKLVDGSSLAAAVVANSIPQGTDQVVLAGNISKVARAVATALCKNNIKVIMSNKQDYHFLKPKIPEDAADNLILSKTSIAKVWVIGEGLDTAEQFRAPKGTHFIPYSPFPPRAVRKDCCTYSTTPAMGVPKTLQNVHSCENWLPRRVMSAWRIAGIIHALEGWNEHECGDTVLDMDKIWSAAILHGFCPVAQA |
| *BdCER1-6* | MASKPGPLTRWPWHDLGNYKYALVAPWAAYSTYSFVAASRRGAQGDLLSFLVLPALLLRLLYTQLWISVSRHQTARSKHRIVNKSLDFDQVDRERNWDDQIILTALLFYVVNATVPMTQGLPWWNSKGLLVTALLHVGPVEFLYYWFHRALHHHFLYSRYHSHHHASIVTEPVTSVIHPFAEEAVYFTLFAIPLLSTMATGTASVAMANGYLVYIDFMNYLGHCNFELVPKLLFDLFPPLKLLMYTPSFHSLHHTQFRTNYSLFMPLYDYVYGTMDKSSDDLYERTLHGREEDRPDVVHLTHLAAPESVLQLRLGFASLAAAPLAFSSSLPGALWTRPLVALASALGRGQAFRSEANRMGKLNAETWVVPRYSSQYTTDVYGVSRLIEKAVSDAEASGAAVLTLGLLNQGYELNRNGELYVIRNPGLKTKIVDGTSLAVAAVLHMIPQGAKDVLLLGKPNKVVSVLALTLCEREFQVGVVDEELHDALRSQLRPEMQRRLVLQPRNYGSKVWLVGDGLTGRDQERAQPGVHFVPYSQFPPSRSARQGDCVCHSTPALVVPDSYENLHACENWLPRRVMSAWRAAGIVHALEKWDGHECGDAVTGVDKAWRAALAHGFKPYDAAAIARQG |
| *BdCER1-7* | MAKRPGPLTEWPWKWMGSFKYLVLAPVAVHTAHMLATKGRGGINPAQATILPILLLRMMHSQIWISLSRHQTARRKHIIVDRSLEFEQVDRERSWDDQIIFSGLLSYMAYLAIPNVSLIPVWSTKGAIITALLHIGPVEFLYYWFHRALHHHFLYSRYHSHHHASIVVEPITSTIHPFAEHLVYFLLFAIPTLVPTLMGRGSIIGVLLYLSYVDFMNNMGHCNFELVPKWILKVFPPMKYLMYTPSFHSLHHTQFRTNYSLFMPFYDYIYNTMDNSTDELYERTLKGTEETLDLVHLTHMTSVQSTYHLRIGVASIASKPSDNSVWYMWMILPMAWLSMVLAWVYGSSAFIVESLKLKKFKMQTWAIPRYNFQYGLICERESINSLIEKAILDADGRGVRVLSLGLLNQEKQLNRSGELFTQKYPNLRVRLVDGSGLATAVVLKSIPLETKRVFLCGTSSKVTQAAATTLCERGVQVIMNQKKAYDMLKLQVPERNTIYLKLSSDEIPQIWIGDNIDDMQQRRAQKGTIFVPTSQFPLKKTRKDDCTYLSSPAMKIPEIMQNVHTCENWHPRRVMSAWRIAGMVHALEGWDMHECGDDMMDTEKVWSAAIKHGFIPLTKA |
| *BdCER1-8* | MATNPGILSEWPWKRLGSFKYLVLAPWVVHGCHLAATKGWRELDLGYVAILPSMLLRALHDQAWITVSRLYNARGKRQIVRRGIEFDQVDRERNWDDQIILSAILLLLGAVYLPGGQHLPLWRTDGAVLLALLHAGPVEFLYYWFHRALHHHVLYTRYHSHHHASIVTEPITSVIHPFAELLAYQLLFSIPMITCALTGTASIITFEIYVIYIDFMNNMGHCNFELVPNWLFEWIPPLKYLMYTPSFHSLHHTQFRTNYSLFMPFYDYIYNTMDKSSETLYQDSLKDKNEEKEVDVVHLTHLTSLQSIYHIRPGFAQYASRPYTSMWHLRIMWPVSWLSMVLTWSYGSWFTVERNVMGKLRMQSWAIPRYRFHYGLKQEKEAINDLIEKAISEADKKGTKVVSLGLLNQAHNLNRSGELYLQKYPKLGVRIVDGTSLAAAVVVNSIPKGTNQVILAGNISKVALAVASALCKKNVKVILTNKQDYHSLKPNIPQNSASNLVLSNTDSAKVWVIGEGLDAAEQLKAEKGTQFIPYSQFPPRMVRKDCCTYLTTPAMSVPKTMQNLHSCENWLPRRVMSAWRIAGIVHALEGWKEDECGDTVLDMEKVWSAAVMHGFSPVAHA |
